# Metasurface-based multi-harmonic free-electron light source

**DOI:** 10.1038/s41377-018-0065-2

**Published:** 2018-09-19

**Authors:** Gilles Rosolen, Liang Jie Wong, Nicholas Rivera, Bjorn Maes, Marin Soljačić, Ido Kaminer

**Affiliations:** 1 Department of Physics, Massachusetts Institute of Technology, 77 Massachusetts Avenue, Cambridge, MA 02139 USA; 2 Micro- and Nanophotonic Materials Group, University of Mons, Place du Parc 20, 7000 Mons, Belgium; 3 Singapore Institute of Manufacturing Technology, 2 Fusionopolis Way, Innovis, Singapore, 138634 Singapore; 4 Department of Electrical Engineering, Technion – Israel Institute of Technology, Haifa, 32000 Israel

## Abstract

Metasurfaces are subwavelength spatial variations in geometry and material where the structures are of negligible thickness compared to the wavelength of light and are optimized for far-field applications, such as controlling the wavefronts of electromagnetic waves. Here, we investigate the potential of the metasurface near-field profile, generated by an incident few-cycle pulse laser, to facilitate the generation of high-frequency light from free electrons. In particular, the metasurface near-field contains higher-order spatial harmonics that can be leveraged to generate multiple higher-harmonic X-ray frequency peaks. We show that the X-ray spectral profile can be arbitrarily shaped by controlling the metasurface geometry, the electron energy, and the incidence angle of the laser input. Using ab initio simulations, we predict bright and monoenergetic X-rays, achieving energies of 30 keV (with harmonics spaced by 3 keV) from 5-MeV electrons using 3.4-eV plasmon polaritons on a metasurface with a period of 85 nm. As an example, we present the design of a four-color X-ray source, a potential candidate for tabletop multicolor hard X-ray spectroscopy. Our developments could help pave the way for compact multi-harmonic sources of high-energy photons, which have potential applications in industry, medicine, and the fundamental sciences.

## Metasurfaces: X-rays from electrons hit by laser

‘Metasurfaces’, whose composition and surface geometry vary at scales smaller than the wavelength of light, could become the new tunable sources of high-energy photons such as X-rays and gamma rays. Gilles Rosolen and colleagues at the Massachusetts Institute of Technology, USA, with co-workers in Belgium and Singapore, used simulation studies to explore the possibilities. Their work demonstrates how a pulsed laser could stimulate free electrons traveling close to a metasurface to generate photons with much higher energies than the incident laser light. The emitted frequency spectrum profile could be controlled by changing the metasurface geometry, the energy of the free electrons, and the angle of the applied laser light. The researchers say that the procedure could be developed to build novel tabletop sources of high-energy photons with many potential applications in industry, medicine and fundamental science.

## Introduction

The development of compact, tunable light sources is an extremely coveted goal: on-chip infrared and optical sources are sought after for photonic integration^1^, while powerful tabletop UV to X-ray sources are potentially useful in applications ranging from medical therapy and diagnostics to industrial quality control, security scans, and the fundamental sciences^2–5^. Passive compact radiation sources based on free electrons have been studied in schemes such as Smith–Purcell emission^6–9^, collective-mode metamaterial light sources^10^, and Cherenkov radiation^11,12^, but are limited by the material response at X-ray frequencies. Recently, active graphene plasmon-based light sources^13,14^ have been considered, exploiting the strongly confined plasmonic field sustained by graphene to generate high-frequency radiation by scattering free electrons. These plasmon-based free-electron light sources have the potential to access extremely high photon energies (e.g., hard X-ray photons) without using highly relativistic electrons or high-intensity lasers. Here, we present a novel plasmonic design that can further reduce the required electron energy as well as generate multi-harmonic X-ray radiation with spatiotemporal profiles that can be directly shaped at the source by the user. More importantly, we show how the concept is not specific to plasmonic designs and generally can be achieved by shaping the near-field of nanopatterned surfaces of any kind. Specifically, we leverage the presence of high-order spatial harmonics in metasurface-enhanced plasmon polaritons that are in turn excited by ultrashort laser pulses to generate multiple higher-order X-ray harmonics via electron–polariton scattering. We show that the metasurface geometry can be tailored to control and optimize the properties of the higher-order spatial harmonics, resulting in a tunable source of multi-harmonic radiation. The ability to generate and tailor multi-harmonic X-ray output is crucial to investigating electron transition dynamics^3,15^, controlling the charge migration in molecules^16,17^, and suppressing photoionization in nonlinear extreme ultraviolet (XUV) spectroscopy^18,19^. Multi-harmonic X-ray sources are also useful in imaging techniques such as multiple-wavelength anomalous diffraction, which determines the different atomic constituents of heterogeneous chemical compositions^20^. The higher-order harmonics also allow a given output photon energy to be accessed at a lower electron energy, paving the way to the realization of compact light sources.

Metasurfaces are metallic or dielectric arrays with subwavelength periodicity and subwavelength thickness that can be used to control the far-field characteristics of incident light^21–23^. This control allows for the observation of a broad variety of phenomena, including anomalous reflection and transmission, perfect lenses, negative refraction, and optical cloaking^21,24–26^. With rapid improvements in fabrication techniques, metasurfaces containing features as small as 10 nm are already attainable via electron beam lithography^27,28^, with a record of sub-5 nm resolution for metallic features^29^. We show that the near-fields of these short-period metasurfaces can be used to realize tunable, multi-harmonic, and highly directional sources of high-energy photons when excited by short laser pulses. The presence of a metasurface enhances the higher-order spatial harmonics of a near-field, leading to polaritons with a distribution of high momenta beyond the momentum of the fundamental mode. These polaritons scatter incident electrons into multiple photon harmonics, whose intensity and other properties depend on the spatial profile of the near-field. The near-field is in turn directly controllable through the metasurface geometry. Throughout our study, we use incident laser pulses with amplitudes of 1 GV/m and pulse durations of 10 fs on a silver metasurface, corresponding to a fluence of 1.4 mJ/cm², which is well below the damage threshold of the silver metasurface^30^. Graphene metasurfaces are illuminated by a laser pulse with an amplitude of 0.1 GV/m^31,32^. We obtain semi-analytical expressions for the radiation output from this interaction, which is a powerful tool for the design of multi-harmonic radiation sources. Our analytical expressions are in excellent agreement with the results of our ab initio numerical simulations.

In the following, we present a concept for a multi-harmonic X-ray source, where electron energy and metasurface geometry can be used to directly tailor the spatiotemporal profile of the X-ray output. We propose an innovative method to sidestep the tradeoff between the interaction length and laser intensity dictated by the damage fluence threshold of a material: by changing the incidence angle of a few-cycle laser pulse so that a high intensity and long interaction length can be simultaneously achieved. By obtaining fully analytical expressions for the intensity of the X-ray output, which we verify with the results of our ab initio simulations, we present an effective and computationally efficient method for optimizing the metasurface profile to produce X-ray output for an arbitrary application. In particular, we present an example for the design of a four-color X-ray source, which has potential applications in spectroscopy and imaging techniques. We also present the concept of a metasurface-based X-ray source powered by a direct current (DC) source instead of a few-cycle laser pulse and compare the features of the two schemes.

## Results

### Metasurface-enhanced plasmon-based sources of high-frequency radiation

**Fig. 1:**
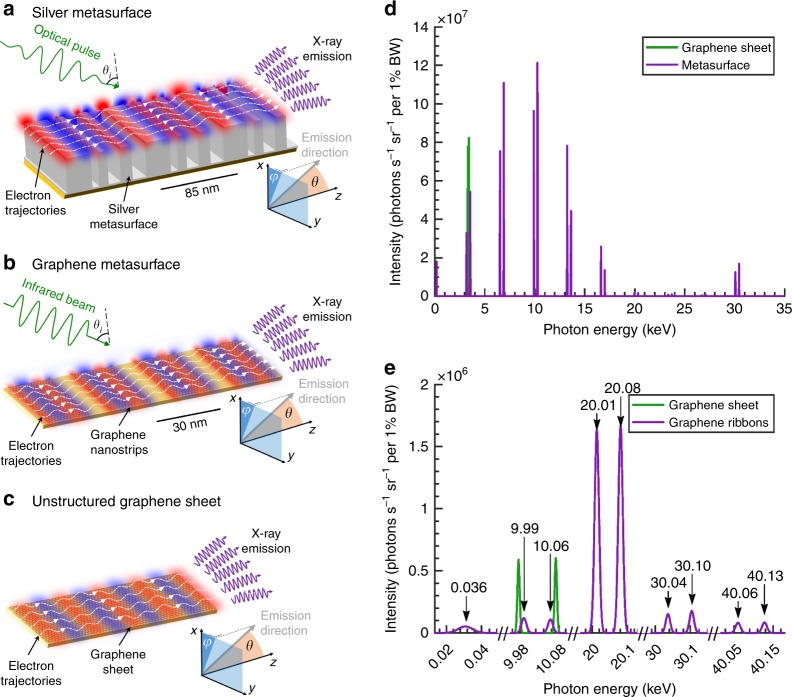
Higher-harmonic X-rays from metasurface-enhanced plasmon-based free-electron light source: The presence of higher spatial harmonics in the near-field profiles leads to output of higher-order X-ray harmonics, allowing the spatiotepmoral characteristics to be tailored via the metasurface geometry. **a** Plasmon polaritons (red and blue) of a silver metasurface with 85-nm periodicity are excited by an optical pulse with a wavelength of 370 nm (3.4 eV) and a peak field amplitude of *E*_0_ = 1 GV/m. An electron beam with a kinetic energy of 5 MeV and an average current of 100 µA is sent parallel to the metasurface at a distance of 1 nm. These electrons are modulated by the plasmonic field, resulting in undulating electron trajectories (dashed white lines) that generate highly directional photons. **b** Plasmon polaritons of a 20-nm wide graphene nanoribbon array (with periods of 28.5 nm) excited by an infrared Gaussian beam with a wavelength of 2 µm and a field amplitude of *E*_0_ = 0.1 GV/m. **c** The process in (**a**) and (**b**) is compared to the unstructured graphene sheet in ref.^13^ using a plasmon wavelength of 85 nm. **d** The sinusoidal trajectory of the electrons modulated by the graphene plasmons (situation (**c**)) results in a single harmonic (green lines), whereas the nonsinusoidal trajectory of the electrons in the metasurface near-field (situation (**a**)) results in multiple monoenergetic harmonics in addition to the free-space Compton harmonic at 0.3 keV and the fundamental harmonics at 3.1 keV and 3.7 keV (purple line). **e** The 5-MeV electron beam with an average current of 100 µA generates multiple harmonics dominated by the second harmonic at a photon energy of 20 keV (situation (**b**)). In both cases, the angle of incidence for the laser is *θ*_*i*_ = 50°

The mechanism for metasurface-mediated electromagnetic radiation with silver and graphene metasurfaces is illustrated in Fig. 1a–c. All the metasurfaces have translational symmetry in the *y*-direction and rest on a glass substrate (*n* = 1.5); we detail the dimensions of the silver metasurface and the optimizations in the Methods section. A laser pulse excites the localized surface plasmon polaritons, which in turn modulate the externally injected electrons, whose undulating trajectories (denoted by the dashed white lines) produce multi-harmonic X-rays. In Fig. 1, we set the laser incidence angle to 50°, which ensures a reasonable interaction length of 18 µm and the central wavelength of the pulse to 370 nm, which corresponds to the plasmonic resonance of silver^33^. Figure 1d compares the radiation produced with the metasurface (Fig. 1a) and that with the graphene sheet proposed in ref.^13^ (Fig. 1c), with fundamental periods of 85 nm for each case. We see that the higher-order spatial harmonics of the plasmonic near-field of the metasurface facilitate the generation of hard X-rays with energies as high as 30 keV, whereas the design of Fig. 1c produces only the first harmonics at 3.1 and 3.7 keV. The kinetic energy of the electrons used in this example is 5 MeV, which is within the range of the state-of-the-art tabletop RF guns^34^. Similarly, Fig. 1e shows that the use of graphene nanoribbons produces multi-harmonic X-rays and enables the access of higher X-ray photon energies than when an unstructured graphene sheet is used.

Each peak in the output spectra of Fig. 1d, e can be traced to distinct spatial harmonics in the plasmonic near-field of the metasurface. For an incident laser pulse with an angular frequency *ω*_0_ (wavelength *λ*_0_) striking the metasurface at an angle *θ*_*i*_, each near-field order *n* creates an emission of order *n* with a frequency defined by

1 $$\omega _{\rm{ph,n}} = \omega _0\left| {\frac{{1 - \beta \left( {\sin \theta _i + \frac{{\lambda _0}}{L}n} \right)}}{{1 - \beta \cos \theta }}} \right|$$

where *θ* is the polar angle of the emitted photon, *L* is the period of the metasurface, and *β* = *v*/*c* is the speed of the electron normalized by *c*, the speed of light in free space. Equation 1 can be directly derived from first principles, using conservation laws (for a walk-through, see the Supplementary Information (SI) section S1). For example, the output spectrum of the 85-nm periodic silver metasurface in Fig. 1b contains at least nine noticeable orders. The peak corresponding to *n* = 0 appears at ℏω_ph,0 _= 0.19 keV and corresponds to the electrons interacting with the sum of the incident and reflected photon field. The higher orders *n* = ± 1, ± 2, … (ℏω_ph,+1  _= 3.2 keV, ℏω_ph,−1  _= 3.6 keV, etc.) correspond to the interactions between the electrons and the higher-order spatial harmonics of the plasmon field, which implies that the higher-order harmonics of the radiation output can be controlled by tailoring the material composition and geometry of the metasurface.

Note that the nonlocal optical responses of the considered metasurfaces are negligible in our regime of interest. According to the hydrodynamic model, a first-order correction of the Drude model^35^, the nonlocal contributions to the metal permittivity are negligible when *k* = *nk*_*L*_ = 2*πn*/*L* ≪ *ω*_0_/*v*_F_, where *v*_F_ is the Fermi velocity. That condition is satisfied for all the output harmonics shown in this paper. For instance, the 9th order of the 85-nm periodic metasurface in Fig. 1d leads to *k* = 6.7·10^8^ rad/m, well below *ω*_0_/*v*_F_ = 3.7·10^9^ rad/m.

### Dynamic tunability of radiation output via electron energy

**Fig. 2:**
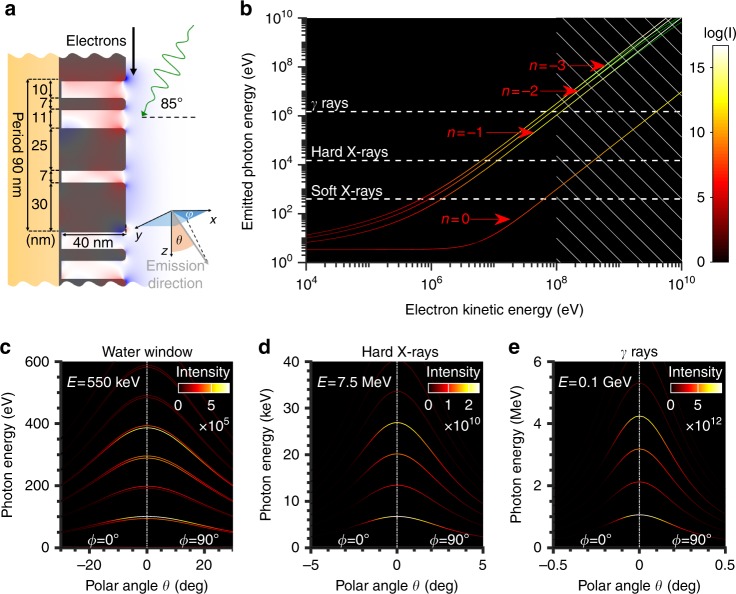
Tunability of the emitted photon energy via the electron energy. **a** Metasurface optimized to enhance the first five orders. The plasmon polaritons are excited by a laser pulse with an energy of 3.4 eV (370 nm), and electrons are shot parallel to the metasurface at a distance of 1 nm. The emission direction is described by *φ* and *θ* in the bottom right inset. **b** At a fixed electron energy, the on-axis output photon energy is higher for higher orders (for visibility, we only present orders *n* = 0, −1, −2, −3) and the source is brighter (intensity in units of number of photons/s/sr/1%BW). At high energies (denoted by the hatched region), our analytical formulation begins to differ from the results predicted by energy–momentum conservation (denoted by green lines), since the analytical formulation does not take quantum recoil into account. **c**–**e** Highly monochromatic harmonic peaks (fractional bandwidths (**c**) < 0.4% (**d**) < 0.2% (**e**) < 0.19%) for **c** soft X-rays from 550 keV electrons (already accessible with a TEM), **d** hard X-rays from 7.5-MeV electrons (can be obtained using a tabletop setup), and **e** *γ*-rays from 0.1-GeV electrons. Note that the line thickness has been artificially increased for clarity

Figure 2 shows the strong influence of the electron's kinetic energy on the radiation frequency, directionality, and intensity (number of photons per second per unit of solid angle per 1% bandwidth) for the metasurface depicted in Fig. 2a. We optimize our metasurface with a fixed periodicity (90 nm) by varying the widths of the three silver blocks that constitute the unit cell and their respective separations and maximize the intensities of the first five orders of the output spectrum (more details in the Methods section). To simplify the analysis, all electrons travel 1 nm above the metasurface in the direction denoted at an average current of 100 µA. A study considering an electron beam with a finite width of 34 nm centered 1.5 nm above the metasurface is conducted in SI section S3. Such electron beams emit radiation at the same frequency and bandwidth as those from the simplified case of a fixed electron trajectory, only with slightly reduced intensity (with an error below 13%, well within an order of magnitude). Figure 2b shows that increasing the energy of the electrons increases the frequency and the intensity of the emitted photons. Additionally, compared with Compton scattering in free space (*n* = 0), electron–polariton scattering using metasurface plasmons can yield photons of the same energy from the electrons, with kinetic energy as much as two orders of magnitude lower. This phenomenon can also be inferred from Equation 1, where we see that decreasing the period *L* and increasing the harmonic number *n* can effectively attain a certain photon energy using much less energetic electrons, thereby removing the need for additional electron acceleration stages.

The radiation spectra in specific emission ranges (water window, hard X-rays, and γ-rays) are shown in Fig. 2c–e. For example, higher near-field orders produce water window photon energies (300–500 eV) with 550-keV electrons, which are already obtainable with a transmission electron microscope (TEM). In addition, 20.2 and 34 keV photons with high directionalities (0.6 and 0.4 mrad angular spread, respectively) are emitted using 7.5-MeV electrons. Notably, the intensity peaks for the various X-ray harmonics are slightly off-axis (i.e., they do not occur exactly at *θ* = 0, as for inverse Compton scattering in free space). This phenomenon is due to the presence of longitudinal electric fields in the near-fields of the metasurfaces, which lead to electron oscillations in the longitudinal and transverse directions. The directionality of the emitted X-ray depends on the electron energy. The angular spread of photon emissions from relativistic electrons scales as γ^−1^ due to relativistic compression of the emission space in the forward direction. When 7.5-MeV electrons are used, an additional acceleration stage after the RF gun may be required, but the entire apparatus could remain tabletop. Importantly, the electron energy is less than 10 MeV, meaning the device does not need additional bulky, heavy neutron shielding^36^.

At higher electron kinetic energies (in excess of 0.1 GeV), the emitted photon energy is close to the electron kinetic energy, and quantum effects need to be considered. Supposing an elastic collision between a photon of momentum $\overrightarrow {p_i} = \hbar \overrightarrow {k_0} + \hbar \overrightarrow {k_L} n$ (where *k*_*L*_ = 2*π*/*L*) and an electron parallel to a metasurface with rest mass *m* and velocity *β* (Lorentz factor *γ* = (1-*β*^2^)^1/2^, the output photon frequency is then given by

2 $$\omega _{\rm {ph,n}} = \omega _0\frac{{\left[ {1 - \beta \left( {\sin \theta _i + \frac{{\lambda _0}}{L}n} \right)} \right] - \frac{{\lambda _0}}{L}n\frac{{2\hbar \omega _0 + k_Lcn}}{{2\gamma mc^2}}}}{{\left( {1 - \beta \cos \theta } \right) + \hbar \omega _0\frac{{1 - {\mathrm{sin}}(\theta + \theta _i)}}{{\gamma mc^2}} - \frac{{\hbar k_Lnc\cos \theta }}{{2\gamma mc^2}}}}$$

The result, represented in Fig. 2b by green lines, deviates from Equation 1, which does not take quantum corrections into account. The deviation is especially pronounced at higher electron energies. At lower electron energies, Equation 2 reduces to Equation 1 when we neglect the effects of quantum recoil (*γmc*^2^ ≫ ℏ*k*_*L*_*nc* ≫ *ℏω*_0_).

We obtain an semi-analytical expression for the on-axis output intensity for each emission order as

3 $$\frac{{d^2I_n}}{{d\omega d{\mathrm{\Omega }}}} \approx \frac{{q^4}}{{32\pi ^2m^2c^2\gamma ^2}}\frac{{\left| {c_{zn}\left( {{\mathrm{\Delta }}x} \right)} \right|^2}}{{\left( {1 - \beta } \right)^2\left( {1 - \beta \sin \theta _i} \right)^2}}T_0^2E_0^2$$

for orders *n* > 0 and

4 $$\frac{{d^2I_0}}{{d\omega d{\mathrm{\Omega }}}} \approx \frac{{q^4}}{{32\pi ^2m^2c^2\gamma ^2}}\frac{{\left| {c_{z01}\left( {{\mathrm{\Delta }}x} \right) - c_{z02}\left( {{\mathrm{\Delta }}x} \right)} \right|^2}}{{\left( {1 - \beta } \right)^2\left( {1 - \beta \sin \theta _i} \right)^2}}\frac{{\left( {\beta - \sin \theta _i} \right)}}{{\cos ^2\theta _i}}T_0^2E_0^2$$

for order *n* = 0, where we assume free space above the metasurface and relativistic electrons (i.e., *β* ≈ 1). The general analytical expressions for the general emission angle are developed in SI section S1. *c*_*zn*_, *c*_*z01*_, and *c*_*z02*_ are the Fourier amplitudes of the electric field along *z* at distance *x* from the metasurface for order *n*, the incident wave and the reflected wave, respectively. *E*_0_ is the incident field amplitude, *T*_0_ is related to the FWHM pulse duration *T*_FWHM_ by the relation *T*_FWHM_ = 2(ln 2)^1/2^*T*_0_, and *θ*_i_ remains the photon incident angle. For our case, the high incidence angle *θ*_i  _= 85° leads to sin *θ*_i_ ≈ 1, resulting in a dependence of the output intensity on the electron velocity that scales as *γ*^−2^(1−*β*)^−4^ ~ γ^6^ for all orders in the relativistic regime, except for order *n* = 0, which is drastically reduced by the factor (*β*−sin *θ*_*i*_)^−2^. This difference directly results in the metasurface-mediated radiation (*n* > 0) being brighter (in addition to being more energetic) than the electron–photon scattering in free space (*n* *=* 0), a property reflected in the output intensities for different orders in Fig. 2, for instance.

### X-ray intensity enhancement via velocity matching

**Fig. 3:**
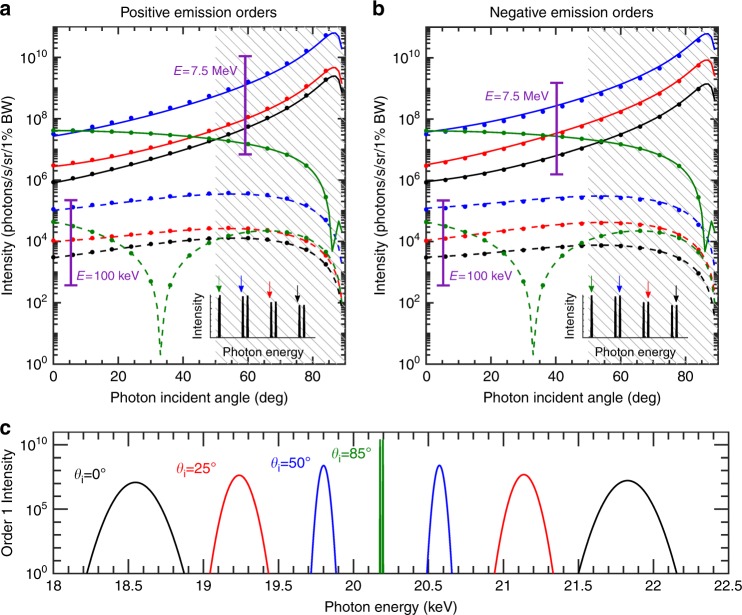
Enhancing X-ray intensity by velocity matching the laser intensity peak and electrons. The maximum intensity for positive and negative emission orders are presented in (**a**) and (**b**), respectively. The inset shows the output spectrum for *θ*_*i*  _=0, and the arrow colors are used to distinguish the different emission orders: order 0 (green), order 1 (blue), order 2 (red), and order 3 (black). Solid (dashed) lines represent the 7.5-MeV (100 keV) electron energy and the dots are approximated in Equations 3 and 4. The logarithmic spectrum in (**c**) shows the peak intensities and energies for the ± 1 emission orders at different angles of incidence of the laser pulse. The metasurface considered has a period of 30 nm consisting of silver blocks 15 nm in width and 40 nm in height laying on a substrate with a refractive index of 1.5, and the spectrum is measured in the direction of the electrons

The intensity of the emitted radiation is also strongly affected by the angle of incidence of the laser pulse, as shown in Fig. 3. With 7.5-MeV electrons, the output intensity increases by three orders of magnitude as the incidence angle is varied from normal incidence *θ*_*i*_ = 0° to the optimal angle *θ*_*i*_ ≈ 86°. With 100-keV electrons, the output intensity varies by one order of magnitude from 0 to the optimal angle *θ*_*i*_ ≈ 55°. The influence of the angle of incidence on the output intensity is attributed to the time of interaction between the electrons and light. Indeed, the speed at which the intensity peak of the pulse travels across the grating is superluminal (*c*/sin*θ*_*i*_). Hence, increasing the incident angle decreases the relative speed of the pulse peak with respect to the electrons, extending the duration of the electron–polariton interaction, an effect directly captured by the term (1−*β* sin*θ*_*i*_)^−2^ in Equation 3. However, the intensity does not peak at *θ*_*i*_ *=* 90° because the *z*-component of the electric field |*c*_zn_| falls sharply at higher angles. The dip in magnitude for the 0th order seen in Fig. 3 (*θ*_*i*_ ≈ 30° for 100-keV electrons) occurs when the electrons have the same velocity as the phase velocity of the photons in the projection parallel to the metasurface. This exact tracking of the phase of the laser by the electrons prevents the laser field from modulating the electrons, suppressing the radiation emitted. This effect is reflected in the factor (*β* − sin *θ*_*i*_)^2^ in Equation 4.

Velocity matching between the electrons and the near-field intensity peak increases the laser intensity without damaging the metasurface, thereby obtaining more intense radiation output. Furthermore, by increasing the laser intensity (|*E*_0_|^2^), the pulse duration (*T*_0_) can be shortened to keep the laser fluence (proportional to |*E*_0_|^2^*T*_0_) below the damage threshold of the metasurface without affecting the duration of the interaction. Throughout our study, we use incident laser pulses with an amplitude of 1 GV/m and a pulse duration of 10 fs, corresponding to a fluence 1.4 mJ/cm², which is well below the damage threshold of the silver metasurface^30^, estimated to be 6.6 mJ/cm² (the details of the calculation are provided in SI Section S5). Similar fluences have been used in the experimental demonstrations of various effects of metasurfaces^30,37^. Studies have also shown that by using appropriately structured elements or more resilient plasmonic materials, the damage threshold can even exceed 100 mJ/cm²^38,39^, allowing us to scale up the X-ray output by increasing the electromagnetic field strength even further. An unstructured graphene sheet supports a strong field of 3 GV/m;^31,32^ however, the edges of nanoribbons present strong field enhancements (~30). Hence, we fix the field amplitude of the Gaussian beam to 0.1 GV/m for the *graphene* metasurface. Another potential means of increasing the interaction time between electrons and photons is the use of tilted-pulse-front pulses^40^, configured such that the intensity peak of the input pulse precisely tracks the progress of the electron bunch across the metasurface.

As Fig. 3c shows, increasing the angle from *θ*_*i*_ = 0° to *θ*_*i*_ = 85° also leads to significant narrowing of the bandwidth as a result of the longer interaction time. Specifically, the fractional bandwidth is narrowed from 0.7% at a central photon energy of 18.5 keV to 0.004% at a central photon energy of 20.18 keV. Additionally, the frequency peaks within each pair of peaks (which correspond to emission orders that are opposite in sign) move closer to each other. That frequency separation can be expressed as 2*ω*_0_(1−*β*sin*θ*_*i*_)/(1−*β*cos*θ*) from Equation 1 and is therefore independent of the order considered.

Varying the angle of incidence of the laser is therefore a convenient way to tune the intensity and the bandwidth, assuming that the electrons remain in the plasmonic near-field throughout the interaction. Although the ponderomotive force exerted by the plasmonic field is negligible at these electron energies^13^, the electron beam divergence could cause the electrons to either significantly move away from the metasurface or hit the metasurface before the end of the interaction, leading to a lower-intensity radiation output and broader bandwidth. The results for electrons with a deflection angle of 0.01° are presented in SI section S4, and the hatched regions in Fig. 3a, b represent the zones where the error of the intensity is more than 50%. To minimize the electron beam divergence due to the space charge, single-electron pulses with high repetition rates can be considered^41,42^. This type of pulse is likely to be feasible in the near future, given that single-electron sources of relativistic energies up to 5 MeV have been recently demonstrated^43^.

### Multi-harmonic output radiation control via metasurface geometry and input wavelength

**Fig. 4:**
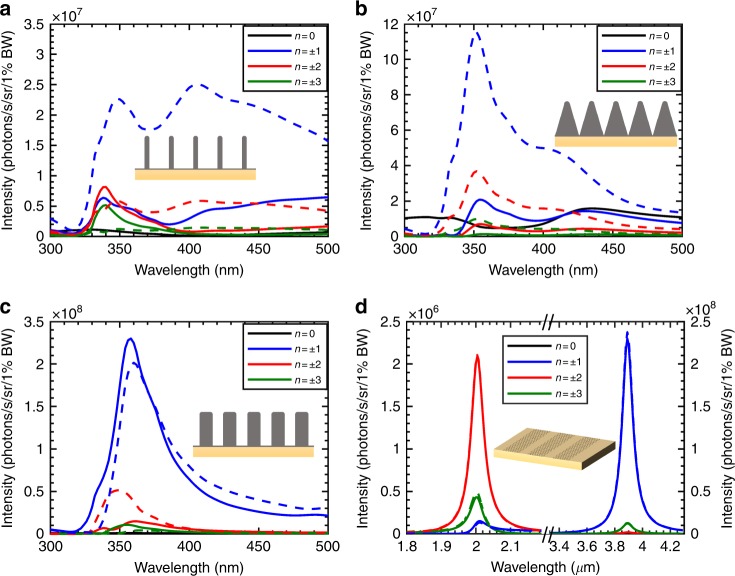
Control of multi-harmonic output radiation via metasurface geometry and input wavelength. In the plots of output intensity as a function of input laser wavelength for various metasurface designs, we see that the silver tips (**a**) and triangular grating structure (**b**) enhance the negative orders (denoted by dashed lines) compared with the positive orders (denoted by solid lines), as opposed to the rectangular grating structure (**c**), where positive and negative orders are much more comparable in intensity. **d** Using the first (second) plasmon mode of the graphene nanoribbon leads to an enhanced first (second) order emission. The silver metasurface with periods of 30 nm and containing 40-nm high structures ((**a**) to (**c**)) and 30- nm periods of 20- nm-wide graphene nanoribbons (**d**). The incident Gaussian beam has an angle of *θ*_i_ = 65°, a spot size of 12 µm FWHM on the metasurface, and an energy of 7.5 MeV. The tips in (**a**) are 6 nm wide and the blocks in (**b**) are 15 nm wide. The incident fields in(**a**)–(**c**) are *E*_0  _= 1 GV/m, and the field in (**d**) is *E*_0  _= 0.1 GV/m

Figure 4 explores a variety of metasurfaces and nanopatterned surfaces available to control and shape X-ray radiation: from nanotips (Fig. 4a) to nanoribbons (Fig. 4d) to periodic grating structures of various shapes (Fig. 4b, c). The intensities of the different emission orders are represented as a function of the wavelength of the incident light. Our plasmonic near-fields are not necessarily the optimal choice of material, e.g., all dielectric metasurfaces create similar physics through their near-field distributions.

Here we consider a continuous wave Gaussian beam, as opposed to the short-pulse excitation of the case considered above. Metasurfaces from Fig. 4a–c show strong plasmonic enhancements around wavelengths of 370 nm, which expectedly coincide with the plasmonic resonance of silver. Furthermore, we see that the metasurface profile plays a key role in defining the near-fields of these structures and, therefore, the intensity of the emission orders. First, the overall intensity is smaller for the tips (Fig. 4a) because the coupling with the incident plane wave is less efficient at the incidence angle of *θ*_*i*_ = 65° chosen for this figure. Indeed, the best coupling for an isolated tip occurs when the electric field is parallel to it, i.e., *θ*_*i*_ = 90°^44^. Second, the geometry of the metasurface influences the near-field profile and therefore the near-field-order amplitudes. For instance, the tips (Fig. 4a) and the triangular gratings (Fig. 4b) favor the negative orders (dashed lines) over the positive orders (solid lines), whereas for the rectangular grating structure (Fig. 4c), they are equivalent. This equivalence can be attributed to the confinement of the field in between the corners of the blocks in the rectangular grating structure, whose profile is less sensitive to the plane wave incident angle than the half-cylindrical shapes of the tips and the triangular structure, where the fields are essentially dipoles that align with the incident plane waves. Further control of the emitted harmonics is possible by engineering asymmetric metasurface elements to tailor the magnitude of the difference between the positive and negative orders within each pair of peaks in the output spectrum. For instance, a blazed grating with different blaze angles is investigated in the SI section S6, where we show that changing the blaze angle from −0.6 to 0.6 rad converts the output spectrum from one that contains dominant negative orders to one that contains dominant positive orders.

The output emission frequency can be dynamically tuned by adjusting the wavelength of the incident light. Specifically, consider switching between excitations of resonances of different orders that are supported by the metasurface to excite higher-order near-field harmonics that directly double (or triple, etc.) the frequency of the emitted light, as we see in the graphene nanoribbon array case of Fig. 4d. With 7.5-MeV electrons, changing the laser source wavelength from 3.9 to 2 µm switches the X-ray emission spectrum from one that peaks at 20 keV (first-order mode) to one that peaks at 40 keV (second-order mode). Equivalently, at a fixed laser wavelength, the switch from first-order to second-order emission modes can be made by an adjustment of the graphene doping^45–49^.

### Multicolor hard X-ray source

The radiation output strongly depends on the distance of the electrons from the metasurface. This dependence is especially challenging when a relatively low periodicity (e.g., 30 nm) is used, due to the strong lateral decay of the strongly confined plasmonic near-field. However, it is possible to enhance the intensities of specific emission harmonics by optimizing the metasurface profile for a given beam distribution through the use of our analytical tools that are in excellent agreement with our ab initio calculations.

**Fig. 5:**
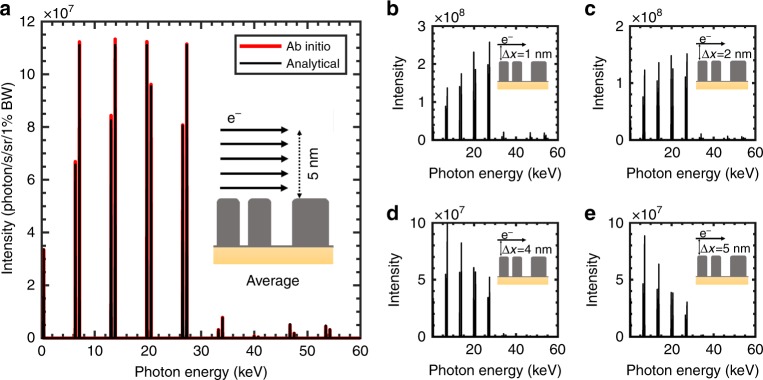
Optimized multicolor hard X-ray source with analytical formulations, showing excellent agreement with ab initio simulations. **a** The average intensity spectrum for a uniformly distributed electron beam, optimizing the metasurface profile with a procedure in which only five-test particle trajectories have to be considered. These five test particles are spaced between 1 and 5 nm away from the metasurface. The optimization results for the five-test particle model show excellent agreement with ab initio simulations of the actual multiparticle beam. The inset shows a period of the metasurface with the electron beam. **b**–**e** The intensity spectra of electrons launched 1 nm (**b**), 2 nm (**c**), 4 nm (**d**), and 5 nm (**e**) from the top of the metasurface. The electron beam energy is 7.5 MeV with an assumed current of 100 µA; the 10-fs laser pulse impinges the metasurface at *θ*_i_ = 50°

Figure 5a illustrates the process of such an optimization, using the goal of a multicolor hard X-ray source as an example. We optimize our metasurface with fixed periods (90 nm) by varying the widths of the three silver blocks that constitute a period and their respective separations, and maximize the intensities of the four first orders of the output spectrum (more details in the Methods section). We successfully design a metasurface using only five test particles in our optimization procedure. The results of our optimization procedure were verified via ab initio many-particle simulations by observing a convergence of results using up to 200 macroparticles. The success of our five-particle optimization process is noteworthy because the behaviors of electron bunches typically require at least 100 particles to simulate accurately, whereas we are able to use only five particles here, leading to significant savings in computational costs.

Figures 5b–e illustrate the dependence of the output spectrum on the electron distance. These figures show that the higher orders decay faster, with electron distance to the metasurface Δ*x* as a result of the *c*_*zn*_ ≈ exp(−*k*_*L*_ *n* Δ*x*) lateral dependence of all the orders. For example, the intensity of the fourth-order emission decays by 90% from the Δ*x* = 1 nm case to the Δ*x* = 5 nm case, whereas the first-order emission decays by only 34%. In Fig. 5a, we consider an elliptical beam 5 nm wide in the *x*-direction and 3 µm wide in the *y*-direction. As before, ab initio many-particle simulations (denoted by the thick red line in Fig. 5a) show excellent agreement with our analytical predictions based on the weighted average of the output spectrum generated by only five test particles (more details in Figure S5). The resulting bright, multicolor spectrum has a peak intensity of 8·10^5^ photons/s in 1% BW (integrating over all angles) for 27-keV photons, which compares with the intensity of 10^6^ photons/s in 1% BW for the high-harmonic generation of 1-keV photons in ref.^50^.

One limitation of the scheme presented is the rapid decay of the near-fields in the transverse direction, making scaling up the X-ray intensity output by increasing the width of the input electron pulse challenging. This rapid decay is the same challenge that confronts dielectric- and metasurface-based laser accelerator designs based on near-field profiles^50–52^. This double-sided metasurface could also be envisaged in our case. To scale up the output X-ray intensity, a multilayer stack could be considered, in which a dielectric medium alternates with the metasurface of choice (e.g., multiple layers of graphene nanoribbons with dielectric spacers or boron nitride heterostructures in between)^53^. In this case, the choice of the dielectric medium is critical, since the interaction distance is limited by the depth of penetration of the electrons into the medium.

### DC metasurfaces for compact X-ray generation

Our studies also lead us to propose an alternative design for a metasurface-based free-electron light source realized by a metasurface with a DC field achieved by biasing the metasurface with a DC voltage or creating a periodic magnetic field (potentially by nanofabrication of magnetic materials). This removes the need for input laser excitation at the expense of the ability to control the properties of the output of high-frequency emissions through parameters such as the wavelength and incidence angle of the input laser pulse. Additionally, the spectra obtained with the DC metasurfaces would also be quite different. First, due to the static nature of the field, the emission peaks would not occur in pairs. The *n* = 0 peak would also be absent because of the absence of the incident field. Second, for a mono-periodic grating with a DC electric field, the effective period of the field would be doubled from the laser-induced to the DC case. This increase occurs because negative and positive voltages cannot coexist on conductive elements in the DC case. As a result, the frequency of the X-ray output is halved from the laser-induced to the DC case when everything else is constant. A comparison between our scheme and one based on a DC nanograting is presented in SI section S7 with a graphene nanoribbon array and a silver metasurface.

## Discussion

In this article, we demonstrate the concept of a metasurface-based free-electron radiation source capable of emitting multiple monoenergetic high-frequency harmonics that are highly directional. The sub-wavelength periodicity of the metasurface allows the X-ray regime to be accessed with much lower electron energies than are needed in a free-space Compton process. We show that still harder X-rays (e.g., 10 times the fundamental harmonic) can be accessed using the same electron energy by leveraging the higher-order plasmonic spatial harmonics of the near-field profiles of metasurfaces. More specifically, we exploit the second-order plasmonic resonance of a graphene nanoribbon array to emit hard X-rays with 5-MeV electrons, already accessible with tabletop RF guns.

We show that the hard X-ray range is accessible with a silver metasurface of 85 nm, where the smallest features are 6 nm. Features as small as 10 nm are already attainable via electron beam lithography^21,22^, and a transverse aspect ratio of 5:1 for 9-nm SiCN wire of 5 µm length has been realized using a mask created by electron beam lithography^54^, even reaching aspect ratios of 25:1 for 9-nm silicon nanowire^55^. Moreover, a record pattern transfer with sub-5 nm resolution has been recently achieved^29^. Therefore, while remaining a challenge, we believe that the aspect ratio height:width (7:1) of our proposed metasurface (height of 40 nm) should be realizable today. However, the height of the metasurface is not crucial for high-energy photon generation. For instance, reducing the height to 20 nm (aspect ratio 3:1) reduces the field enhancement by 27%. The harmonic amplitudes of the near-field follow the same trend, but not uniformly. By optimizing the profile of the metasurface and by compensating for the field enhancement drop with the intensity of the driving laser, the proposed X-ray spectra are achievable at reduced metasurface height. We believe that the continued push toward smaller resolutions in nanolithography will bring more tunable sources of high-energy photons within reach at decreasing electron energies.

We develop analytical expressions that are in excellent agreement with the results of our ab initio simulations. We show that the emission intensity and bandwidth can be controlled through various parameters, including the angle of incidence of the laser input, the pulse duration, and the electron kinetic energy, to meet the specifications of a given application. The results we present rely on an electron beam traveling parallel to the metasurface. We show that a misalignment of the electrons prevents the scaling up of the output intensity. However, single-electron sources with high repetition rates are most likely to be feasible in the near future^43^, prefacing negligible beam divergence. However, highly divergent electron beams may be aimed along the metamaterials (e.g., graphene multilayer with dielectric spacer) rather than metasurfaces, allowing all electrons to interact with the plasmonic field.

In Fig. 5, we present a highly directional (emission angle ± 0.5 mrad) and bright (8·10^5^ photons/s/1%BW at the 27-keV X-ray photon peak) multicolor hard X-ray source with multiple monoenergetic peaks (fractional bandwidth less than 0.3%). The intensity of this source is comparable with the intensity of 10^6^ photons/s in 1% BW for high-harmonic generation of 1-keV photons that was recently reported in^56^. Unlike the photons generated in ref.^57^, our photons are at much higher photon energies (e.g., 27 keV). We estimate our source brightness to be 6.7·10^8^ photons/s/mm²/mrad²/0.1% BW, assuming an emission area of 3 µm by 5 nm (equal to the cross-section of the electron beam, since our source is highly directional). This result compares favorably to the X-ray tube, which shows a brightness between 10^5^ and 10^7^ photons/s/mm²/mrad²/0.1% BW in addition to also being tunable and much more directional. Furthermore, engineering the input electron source may scale up the intensity by several orders of magnitude. Periodic prebunching of electrons, which can be performed using diffraction-related methods of electron waveshaping^57^, leads to an enhancement of up to a factor equal to the number of electrons in the bunch^58^. For instance, a similar electron source with an average current of 100 µA composed of 1.6-pC bunches with a repetition rate of 62.5 MHz^59^ can improve the radiation intensity by seven orders of magnitude. Increasing the average current is yet another way of enhancing the output intensity. Recently, a 5-MeV electron beam of 65 mA in current has been presented, and an average current of 100 mA is expected to occur in the near future^59^. Such a current would enhance our source intensity by three orders of magnitude. These enhancements can potentially take the brightness of our source to near-synchrotron brightness levels, which are typically between 10^16^ and 10^18^ photons/s/mm²/mrad²/0.1% BW (and higher for newer generations).

Such a multicolor source, with its highly directional output, monochromatic peaks, and user-tailorable output spectrum, would be extremely useful in time-resolved X-ray spectroscopy for ultrafast imaging of electronic-state transitions^3,15^, to control the charge migration in the molecules^16,17^ and suppress photoionization in nonlinear XUV spectroscopy^18,19^. Further work could include two-dimensional materials^60^, such as hexagonal boron nitride (hBN), supporting highly subwavelength phonon polaritons; these materials suggest exciting possibilities for new and improved structures. Additionally, an output spectrum spatiotemporally shaped via tunable materials like graphene^61^ or reconfigurable metasurfaces can be imagined^62^.

## Materials and methods

The output spectra are computed via ab initio simulations in which the electromagnetic fields are modeled using Maxwell’s equations, and the electron behavior is modeled using the Newton–Lorentz equations of motion without any approximations. Specifically, we use a frequency domain numerical field solver (COMSOL RF Module) to obtain the metasurface response of a plane wave excitation across the range of frequencies in the incident Gaussian light pulse. The resulting electromagnetic fields are used to compute the exact electron trajectories by means of the Newton–Lorentz equation of motion as well as the resulting output radiation spectrum. Our electrodynamics solver takes into account both the near-field and far-field interactions between the electrons. We also derive analytical expressions that show excellent agreement with the numerical results in the regime of interest. The details of the analytical model and a comparison between the analytical and numerical results are provided in the Supplementary Information (SI) sections S1 and S2.

In all the cases involving silver gratings, we consider a short pulse of 10 fs in duration because shorter pulses enable higher-intensity output radiation without damaging the metasurface (see discussion of Fig. 3). Our dispersionless analytical formulation is robust for such short pulses, provided that the plasmonic field enhancement is not locally peaked in frequency. For our silver metasurface, a maximum error of 1% in the complete ab initio calculations is shown in SI S2. In the case of the graphene nanostrips, we consider a continuous wave Gaussian beam with a spot size of 12 µm full-width-at-half-maximum (FWHM) on the metasurface. In all cases, we consider 200 particles for the ab initio simulations, verifying that the simulation results have converged by varying the number of particles while keeping the total current constant. We model the dispersion relation of graphene using the local random phase approximation^63,64^ at room temperature with 0.66 eV doping and scattering lifetime *τ* *=* 0.2 ps, while the silver optical parameters are from ref.^33^. All the silver metasurfaces have translational invariance in *y*, have a height of 40 nm, and rest on a glass substrate where the refractive index*n* = 1.5. The corners are rounded with a curvature radius of 2.5 nm, and residual silver layers sized 2 nm lie in between the blocks to take into account the possible residuals of the electron beam lithography (the presence of that thin layer does not influence the near-field interactions with the electrons). The unit cell of the silver metasurface of Fig. 1a is composed of blocks 18, 26, and 9 nm in width, spaced 9, 9, and 14 nm from each other, respectively. The metasurface considered in Fig. 2 has a period of 30 nm with silver blocks 15 nm wide. The optimized metasurface of Fig. 5 has a period of 90 nm and is composed of three blocks of 18 nm in width and separated from each other by 22, 9, and 5 nm. We consider graphene and silver for their interesting plasmonic properties in the optical and infrared ranges, respectively, but the analytical formulation is also applicable to the study of electromagnetic surface waves on dielectric structures or hybrid (metal–dielectric) structures in general.

The optimization procedure was conducted as follows. First, we fix the period of the metasurface (85 nm in Fig. 1 and 90 nm in other figures), hence, we automatically define the harmonics, i.e., the output frequencies of the spectrum (see Equation 1). Second, we design the shape of the features, forming the unit cell; here, we choose three blocks for simplicity in implementation and fabrication (the choice of the three blocks provides enough degrees of freedom (5) for the optimization while allowing for them to be wide enough to be feasibly fabricated). The widths and the positions of the blocks influence the near-field and therefore the amplitudes of the fields in each Fourier component and, hence, also the amplitudes of the peaks in the output X-ray spectrum. Finally, we optimize the widths and the positions of these three blocks at fixed periods to obtain the desired spectrum. Specifically, in the case of Fig. 5, we maximized the intensities of the X-rays for the four first harmonics by optimizing the width and position of the three blocks under the constraints of 90-nm periods (same optimization as for Fig. 2, but for five orders). The energy of the electron beam was set to 7.5 MeV to reach the hard X-ray range (see discussion of Fig. 2), the angle of the laser was fixed at 50° to ensure a reasonable interaction length (see discussion of Fig. 3), and the wavelength was set at 370 nm to be near the plasmonic resonance of silver (see discussion of Fig. 4).

## Electronic supplementary material

Supplementary Information
