# Supplementary material for: Metasurface-based multi-harmonic free-electron light source
Source: Light Sci Appl. 2018 Sep 19;7:64. doi: 10.1038/s41377-018-0065-2 (PMC6143620; doi:10.1038/s41377-018-0065-2)
Supplement: Supplementary file 1 — Supplementary Information [file 41377_2018_65_MOESM1_ESM.pdf]

Metasurface-based multi-harmonic free electron light source

Supplementary Information

Gilles Rosolen*[1,2,†], Liang Jie Wong[3,†], Nicholas Rivera[1], Bjorn Maes[2], Marin Soljačić[1], and Ido Kaminer[4]

[1]Department of Physics, Massachusetts Institute of Technology, 77 Massachusetts Avenue, Cambridge 02139, Massachusetts, USA

[2]Micro- and Nanophotonic Materials Group, University of Mons, Place du Parc 20, 7000 Mons, Belgium

[3]Singapore Institute of Manufacturing Technology, 2 Fusionopolis Way, Innovis, Singapore 138634, Singapore

[4]Department of Electrical Engineering, Technion – Israel Institute of Technology, Haifa 32000, Israel

[†]Authors with equal contributions

gilles.rosolen@umons.ac.be

Contents

S1. Electron-polariton scattering off plasmonic metasurfaces:
A classical electrodynamic treatment 2

a. Overview 2

b. Continuous-wave fields of a metasurface-enhanced plasmon 3

c. Pulsed fields of a metasurface-enhanced plasmon 6

d. Electrodynamics in the pulsed fields of metasurface-enhanced plasmons 9

e Spectral intensity of emitted radiation 12

f. The case of a continuous-wave laser 15

S2. Comparison with *ab initio* calculations 18

S3. Electron beam spatial extent 21

S4. Electron beam angle dependence 25

S5. Laser fluence 26

S6. Asymmetry of the spectrum enabled by a blazed grating 28

S7. DC fields 29

References 33

S1. Electron-polariton scattering off plasmonic metasurfaces: A classical electrodynamic treatment

a. Overview

The goal of this write-up is to present an analytical approximation of the radiation emitted from an electron passing through plasmonic fields supported by a periodic metasurface structure. Such an expression would be useful not only in predicting the output radiation properties in closed-form, but also in guiding the optimal metasurface design, based on the desired output spectrum.

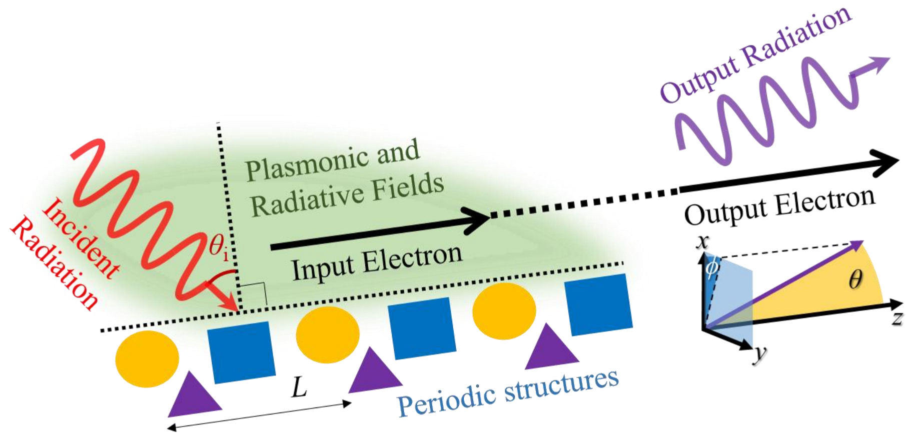

Figure S1: An electron scattering off the electromagnetic fields of a plasmonic metasurface emits photons at different frequencies and angles. The field with which the electron interacts includes induced localized plasmons as well as any incident, reflected, and scattered radiation. In the scenario considered here, the change in energy-momentum experienced by the electron is negligible. The output photon is detected at observation angles (θ, ϕ). The electron is assumed to travel in the +z direction, parallel to the periodicity of the metasurface.

In what follows, we consider the interaction of a charged particle with initial kinetic energy $(\gamma_0 - 1)mc^2$ and z-directed velocity $v_{z0} \equiv \beta_{z0}c$, where c is the speed of light in free space, m the particle rest mass and $\gamma_0 \equiv 1\big/\sqrt{1-\beta_{z0}^{\,2}}$ the initial Lorentz factor. The particle interacts with the field near a plasmonic metasurface that is periodic along the z direction, with period L. This interaction, which is assumed to take place in a linear, isotropic and time-invariant medium, is illustrated in Figure S1.

b. Continuous-wave fields of a metasurface-enhanced plasmon

We begin by considering the response of a metasurface to an incident continuous-wave (CW) laser. The electromagnetic fields supported by a periodic grating structure, assuming excitation by a CW electromagnetic beam of angular frequency ω_0, can be written as

$$\begin{aligned} E_{\{x,y,z\},\mathrm{CW}}(x,z,t,\omega_0) &= \mathrm{Re}\{E_{\{x,y,z\}0}(x,z,\omega_0)\exp(\mathrm{i}k_{z0}z-\mathrm{i}\omega_0 t)\} \\ B_{\{x,y,z\},\mathrm{CW}}(x,z,t,\omega_0) &= \mathrm{Re}\{B_{\{x,y,z\}0}(x,z,\omega_0)\exp(\mathrm{i}k_{z0}z-\mathrm{i}\omega_0 t)\}, \end{aligned} \tag{S1.1}$$

where $k_{z0} = k_0 \sin\theta_\mathrm{i}$, $k_0 = \omega_0/u$ is the wavevector magnitude, u is the speed of light in the medium, and we have assumed that the input beam is incident in the plane containing the dimension of periodicity (here, the z dimension), at an angle θ_i to the normal of the surface. In accordance with the Bloch-Floquet theorem, E_{x0}, E_{y0}, E_{z0}, B_{x0}, B_{y0}, B_{z0} are periodic functions such that

$$\begin{aligned} E_{\{x,y,z\}0}(x,z,\omega_0) &= E_{\{x,y,z\}0}(x,z+L,\omega_0) \\ B_{\{x,y,z\}0}(x,z,\omega_0) &= B_{\{x,y,z\}0}(x,z+L,\omega_0), \end{aligned} \tag{S1.2}$$

where L is the period of the metasurface. By applying a Fourier series decomposition to the fields in (S1.2), we obtain

$$\begin{aligned} E_{\{x,z\}0}(x,z,\omega_0) &= \sum_{\substack{n=-\infty,\\ n\neq 0}}^{n=\infty} c_{\{x,z\}n}\exp[\mathrm{i}(k_{xn}x_r + nk_L z)] + c_{\{x,z\}01}\exp(\mathrm{i}k_{x0}x_r) + c_{\{x,z\}02}\exp(-\mathrm{i}k_{x0}x_r) \\ B_{y0}(x,z,\omega_0)u &= \sum_{\substack{n=-\infty,\\ n\neq 0}}^{n=\infty} c_{yn}\exp[\mathrm{i}(k_{xn}x_r + nk_L z)] + c_{y01}\exp(\mathrm{i}k_{x0}x_r) + c_{y02}\exp(-\mathrm{i}k_{x0}x_r) \\ B_{\{x,z\}0}(x,z,\omega_0)u &= \sum_{\substack{n=-\infty,\\ n\neq 0}}^{n=\infty} d_{\{x,z\}n}\exp[\mathrm{i}(k_{xn}x_r + nk_L z)] + d_{\{x,z\}01}\exp(\mathrm{i}k_{x0}x_r) + d_{\{x,z\}02}\exp(-\mathrm{i}k_{x0}x_r) \\ E_{y0}(x,z,\omega_0) &= \sum_{\substack{n=-\infty,\\ n\neq 0}}^{n=\infty} d_{yn}\exp[\mathrm{i}(k_{xn}x_r + nk_L z)] + d_{y01}\exp(\mathrm{i}k_{x0}x_r) + d_{y02}\exp(-\mathrm{i}k_{x0}x_r) \end{aligned} \quad ,\text{(S1.3)}$$

where $k_{xn} = \sqrt{k_0^{\ 2} - k_{zn}^{\ 2}}$, $k_{zn} = nk_L + k_{z0}$, $k_{z0} = k_0 \sin\theta_{\rm i}$, $k_L = 2\pi/L$, $x_{\rm r} = x - x_0$, where x_0 is an arbitrary reference position. Due to the boundary conditions of the physical problem the only term traveling in the -x direction is the incident field. Note that k_{xn} can be either real or imaginary (if imaginary, the requirement $k_{xn} < 0$ must be met so that fields decay in +x direction, assuming the electron travels above the surface), whereas k_{x0}, k_{L}, k_{z0} and k_{zn} are always real. Typically for metasurfaces that are extremely subwavelengths, as we consider in this work, all the k_{xn} have an imaginary component and represent a decaying near-field. Applying the Maxwell equations to Eq. (S1.3), we find that the complex coefficients are related to one another by

$$\begin{aligned}
&c_{xn} = -\frac{k_{zn}}{k_{xn}} c_{zn},\ c_{x01} = -c_{z01} \tan\theta_{\rm i},\ c_{x02} = c_{z02} \tan\theta_{\rm i}, \\
&c_{yn} = -\frac{k_0}{k_{xn}} c_{zn},\ c_{y01} = -c_{z01} \sec\theta_{\rm i},\ c_{y02} = c_{z02} \sec\theta_{\rm i}, \\
&d_{xn} = -\frac{k_{zn}}{k_{xn}} d_{zn},\ d_{x01} = -d_{z01} \tan\theta_{\rm i},\ d_{x02} = d_{z02} \tan\theta_{\rm i}, \\
&d_{yn} = \frac{k_0}{k_{xn}} d_{zn},\ d_{y01} = d_{z01} \sec\theta_{\rm i},\ d_{y02} = -d_{z02} \sec\theta_{\rm i}.
\end{aligned} \tag{S1.4}$$

As Eq. (S1.4) shows, the coefficients in the expressions for $E_{\rm x}$, $E_{\rm y}$, $B_{\rm x}$, $B_{\rm y}$ can be directly determined from the coefficients in the expressions for $E_{\rm z}$, $B_{\rm z}$. Hence, it is possible to construct the solution of the entire field above the periodic structure from just the z components of the fields. More specifically, the solution can be constructed from only $E_{z0}(x_1, z, \omega_0)$, $E_{z0}(x_2, z, \omega_0)$, $B_{z0}(x_1, z, \omega_0)$, $E_{z0}(x_1, z, \omega_0)$, where $x_{1,2}$ are arbitrary x positions above the grating, and we may restrict the domain to $0 < z \le L$ due to the functions' periodicity in z. These points of data can be obtained from numerical field solvers (e.g., COMSOL). Using the Fourier series relations, we then determine the coefficients c_{zn}, c_{z01} and c_{z02} as

$$c_{zn} = \exp(-\mathrm{i}k_{xn}x_{1r})\frac{1}{L}\int_0^L \mathrm{d}z E_{z0}(x_1, z, \omega_0)\exp(-\mathrm{i}nk_L z) \tag{S1.5.1}$$

and

$$\begin{bmatrix} c_{z01} \\ c_{z02} \end{bmatrix} = \frac{1}{2\mathrm{i}\sin(k_{x0}x_{12})}\begin{bmatrix} \exp(-\mathrm{i}k_{x0}x_{2r}) & -\exp(-\mathrm{i}k_{x0}x_{1r}) \\ -\exp(\mathrm{i}k_{x0}x_{2r}) & \exp(\mathrm{i}k_{x0}x_{1r}) \end{bmatrix}\begin{bmatrix} \frac{1}{L}\int_0^L \mathrm{d}z E_{z0}(x_1, z, \omega_0) \\ \frac{1}{L}\int_0^L \mathrm{d}z E_{z0}(x_2, z, \omega_0) \end{bmatrix}, \tag{S1.5.2}$$

where $x_{1\mathrm{r}} = x_1 - x_0$, $x_{2\mathrm{r}} = x_2 - x_0$, and $\mathrm{x}_{12} = \mathrm{x}_1 - \mathrm{x}_2$. Equivalent expressions for $d_{\mathrm{z}n}$, $d_{\mathrm{z}01}$ and $d_{\mathrm{z}02}$ may be obtained by replacing $c_{\mathrm{z}n}$, $c_{\mathrm{z}01}$, $c_{\mathrm{z}02}$, $E_{\mathrm{z}0}$ with $d_{\mathrm{z}n}$, $d_{\mathrm{z}01}$, $d_{\mathrm{z}02}$, $B_{\mathrm{z}0}$ respectively in Eqs. (S1.5.1) and (S1.5.2). Ideally, x_1 should be chosen close to the nanostructures where the plasmon fields are strong, and x_2 should be chosen a distance away that is at least on the order of the wavelength of the incident free-space radiation, so it captures mostly the incident (and reflected) fields and only a negligible contribution from the plasmons. In this way, the fields supported by a periodic nanostructure may be obtained in a semi-analytical form – Eqs. (S1.3) and (S1.4) – with the help of field solver software to determine coefficients through Eqs. (S1.5.1) and (S1.5.2). Note that although there are in principle an infinite number of coefficients, the fields are dominated only by a few coefficients located at small values of $|n|$ in most realistic scenarios. Eqs. (S1.3) and (S1.4) will be useful in determining a semi-analytical estimate for the radiation spectrum, as is our goal in this section. They can also be used to speed up numerical evaluation of electrodynamics in particle-tracking solvers by reducing the size of the input field from two-dimensional or three-dimensional look-up tables – one for each component of the E and B fields – to just the few dominant coefficients in the $E_{\mathrm{z}0}$ and $B_{\mathrm{z}0}$ Fourier series expansion.

c. Pulsed fields of a metasurface-enhanced plasmon

Next we consider the response of a metasurface to an incident pulsed laser. A pulsed plasmon results when the incident field – captured by the terms in Eq. (S1.3) that contain coefficients with subscript “02” in the previous case – is a pulse instead of a CW plane wave. Whereas the E_x-component of the CW incident field was given in Section *b* by

$$E_{x,\mathrm{in,CW}} = \mathrm{Re}\left\{c_{x02}\exp\left(-\mathrm{i}k_{x0}x_r + \mathrm{i}k_{z0}z - \mathrm{i}\omega_0 t\right)\right\}, \tag{S1.6}$$

we consider here a pulsed version of the form

$$E_{x,\mathrm{in}} = \mathrm{Re}\left\{c_{x02}\exp\left(-\mathrm{i}k_{x0}x_r + \mathrm{i}k_{z0}z - \mathrm{i}\omega_0 t\right)\exp\left[-\left(-\cos\theta_\mathrm{i}\frac{x_r}{u} + \sin\theta_\mathrm{i}\frac{z}{u} - t\right)^2 \Big/ \left(2T_0{}^2\right)\right]\right\}, \tag{S1.7}$$

where T_0 is related to the (intensity) full-width-at-half-maximum pulse duration T_{FWHM} by the relation $T_{\mathrm{FWHM}} = 2\sqrt{\ln 2}T_0$. Note that Eq. (1.7) is a valid approximation of an electromagnetic pulse when $T_0 >> 2\pi/\omega_0$. Choosing too small a pulse duration results in significant DC (i.e. static) components and thus an unrealistic model for a propagating pulse. Comparing Eqs. (S1.6) and (S1.7), we see that we can obtain the overall electromagnetic field corresponding to the input pulse described by Eq. (S1.7) via the equations

$$\begin{aligned} E_{\{x,y,z\}}(x,z) &= \int_{-\infty}^{\infty} E_{\{x,y,z\},\mathrm{CW}}(x,z,t,\omega)G(\omega)\mathrm{d}\omega \\ B_{\{x,y,z\}}(x,z) &= \int_{-\infty}^{\infty} B_{\{x,y,z\},\mathrm{CW}}(x,z,t,\omega)G(\omega)\mathrm{d}\omega \end{aligned}, \tag{S1.8}$$

where

$$G(\omega) = \frac{T_0}{\sqrt{2\pi}}\exp\left[-\frac{(\omega-\omega_0)^2 T_0{}^2}{2}\right]. \tag{S1.9}$$

under the condition $2\pi/L >> k_0$ – which implies that the length L of the fundamental metasurface period is much smaller than the central wavelength of the incident pulse – we can evaluate Eqs. (S1.8) and (S1.9) to obtain the following analytical description of the overall electromagnetic pulse:

$$\begin{aligned}
E_{\{x,z\}}(x,z,t) &= \mathrm{Re}\left\{ \sum_{\substack{n=-\infty,\\ n\neq 0}}^{n=\infty} c_{\{x,z\}n}' g_n f_n + c_{\{x,z\}01} g_{01} f_{01} + c_{\{x,z\}02} g_{02} f_{02} \right\} \\
B_y(x,z,t)u &= \mathrm{Re}\left\{ \sum_{\substack{n=-\infty,\\ n\neq 0}}^{n=\infty} c_{yn}' g_n f_n + c_{y01} g_{01} f_{01} + c_{y02} g_{02} f_{02} \right\} \\
B_{\{x,z\}}(x,z,t)u &= \mathrm{Re}\left\{ \sum_{\substack{n=-\infty,\\ n\neq 0}}^{n=\infty} d_{\{x,z\}n}' g_n f_n + d_{\{x,z\}01} g_{01} f_{01} + d_{\{x,z\}02} g_{02} f_{02} \right\} \\
E_y(x,z,t) &= \mathrm{Re}\left\{ \sum_{\substack{n=-\infty,\\ n\neq 0}}^{n=\infty} d_{yn}' g_n f_n + d_{y01} g_{01} f_{01} + d_{y02} g_{02} f_{02} \right\}
\end{aligned}, \qquad \text{(S1.10)}$$

The variables associated with the pulse envelopes are given by

$$\begin{aligned}
g_{01} &= \exp\left[-\left(\frac{x_r}{u}\cos\theta_\mathrm{i} + \frac{z}{u}\sin\theta_\mathrm{i} - t \right)^2 \Big/ \left(2T_0^{\,2}\right) \right] \\
g_{02} &= \exp\left[-\left(-\frac{x_r}{u}\cos\theta_\mathrm{i} + \frac{z}{u}\sin\theta_\mathrm{i} - t \right)^2 \Big/ \left(2T_0^{\,2}\right) \right] \\
g_n &= \frac{T_0}{T_{0n}'}\exp\left[-\left(\frac{z}{u}\sin\theta_\mathrm{i} - t \right)^2 \Big/ \left(2T_{0n}'^{2}\right) \right] \\
T_{0n}' &= \sqrt{T_0^{\,2} + \frac{x_r\left(3\sin^2\theta_\mathrm{i} - 1\right)}{2\pi n u^2/L}}
\end{aligned} \qquad \text{(S1.11)}$$

The variables associated with the carriers are given by

$$
\begin{aligned}
&f_{01} = \exp\left(\mathrm{i}k_{\mathrm{x0}}x_r + \mathrm{i}k_{\mathrm{z0}}z - \mathrm{i}\omega_0 t\right) \\
&f_{02} = \exp\left(-\mathrm{i}k_{\mathrm{x0}}x_r + \mathrm{i}k_{\mathrm{z0}}z - \mathrm{i}\omega_0 t\right) \\
&f_n = \exp\left(\mathrm{i}k_{\mathrm{xn}}'x_r + \mathrm{i}k_{\mathrm{zn}}'\mathrm{z} - \mathrm{i}\omega_{\mathrm{0n}}'t\right) \\
&\omega_{\mathrm{0n}}' = \omega_0 + \delta\omega_n \\
&\delta\omega_n = -2\frac{x_r}{u}\left[\frac{\omega_0\left(3\sin^2\theta_{\mathrm{i}} - 1\right)}{2\pi nu/L} + \sin\theta_{\mathrm{i}}\right] \Big/ \left(2T_{0n}'^2\right) \\
&k_{xn}' = k_{xn} + \mathrm{i}\frac{\delta\omega_n}{2u}\left[\frac{\omega_0\left(3\sin^2\theta_{\mathrm{i}} - 1\right)}{2\pi nu/L} + \sin\theta_{\mathrm{i}}\right] \\
&k_{zn}' = k_{zn} + \frac{\delta\omega_n}{u}\sin\theta_{\mathrm{i}}
\end{aligned}
\quad . \qquad \text{(S1.12)}
$$

The c and d coefficients in the summation term of Eq. (S1.10) are related by

$$
\begin{aligned}
&c_{\mathrm{x}n}' = -\frac{k_{zn}'}{k_{xn}'}c_{zn}', \;\; c_{\mathrm{y}n}' = -\frac{\omega_{0n}'}{k_{xn}'u}c_{zn}' \\
&d_{\mathrm{x}n}' = -\frac{k_{zn}'}{k_{xn}'}d_{zn}', \;\; d_{\mathrm{y}n}' = \frac{\omega_{0n}'}{k_{xn}'u}d_{zn}'
\end{aligned}
\quad . \qquad \text{(S1.13)}
$$

The conditions under which Eqs. (S1.10)-(S1.13) provide a good analytical approximation for the pulsed plasmon are described by the inequality

$$
L << \frac{2\pi}{k_0} << uT_0 . \qquad \text{(S1.14)}
$$

In obtaining Eqs. (S1.10)-(S1.13), we have also assumed that the effects of material dispersion are negligible, implying that the c and d coefficients in the integrands of Eq. (S1.8) do not change substantially over the bandwidth of the pulse spectrum. We discuss in section S2 the influence of the dispersion on the output radiation. Exactly accounting for the dispersion (i.e. refractive index variation with frequency) involves multiplying each line of the CW response (Eq. (S1.3)) with the

spectral distribution $G(\omega)$ and integrating of the result over ω. Note in this case that each of the c and d coefficients in the integrand are also a function of ω.

Note that the first inequality in Eq. (S1.14) implies that all terms in Eq. (S1.10) describe evanescent plasmonic modes except for the reflected and incoming fields (which are respectively the second-last and last terms in the Re{} function of each line in Eq. (S1.10)). Eq. (S1.14) applies to a broad range of practical problems, including our scenarios of interest where many-cycle pulses of central free space wavelength ~340-4000 nm are incident on metasurface structures of fundamental period ~30 nm.

d. Electrodynamics in the pulsed fields of metasurface-enhanced plasmons

In this subsection, we solve for the motion of electrons interfacing the field near a metasurface. The Newton-Lorentz equation of motion is given by

$$\frac{d\mathbf{p}}{dt} = q(\mathbf{E} + \mathbf{v} \times \mathbf{B}), \tag{S1.15}$$

where $\mathbf{p}$ is the momentum of the particle, q its charge and $\mathbf{v}$ its velocity. $\mathbf{E}$ and $\mathbf{B}$ are the pulsed electromagnetic fields obtained in Section S1c. We operate in the regime where the particle moves mostly parallel to the surface $\forall t$ (i.e., $v_{x,y}/u << 1$ and $\gamma \cong 1/\sqrt{1-\beta_z^2}$), which applies in most realistic cases of interest involving quasi-to-highly relativistic particles. Only extremely high intensities can cause a quasi-to-highly relativistic particle to deviate significantly from the (mostly) parallel motion, however such intensities are usually irrelevant to our problem anyway, due to damage threshold restrictions of the metasurface material. Under such (mostly) parallel motion, we find that the velocity and position of the charged particle may be approximated by the following equations

$$v_x \cong \frac{\mathrm{i}q}{2m\gamma_0}\left\{\sum_{\substack{n=-\infty,\\ n\neq 0}}^{n=\infty}\frac{k_{zn}'-v_{z0}\omega_{0n}'/u^2}{k_{xn}'\left(k_{zn}'v_{z0}-\omega_{0n}'\right)}c_{zn}g_n\left(v_{z0}t,t\right)\exp\left[\mathrm{i}\left(k_{xn}'x_r+\left\{k_{zn}'v_{z0}-\omega_{0n}'\right\}t\right)\right]\right.$$
$$+\frac{\tan\theta_{\mathrm{i}}-\left(v_{z0}/u\right)\sec\theta_{\mathrm{i}}}{k_{z0}v_{z0}-\omega_0}\left[c_{z01}g_{01}\left(v_{z0}t,t\right)\exp\left(\mathrm{i}k_{x0}x_r\right)\right.\quad\text{, (S1.16.1)}$$
$$\left.\left.-c_{z02}g_{02}\left(v_{z0}t,t\right)\exp\left(-\mathrm{i}k_{x0}x_r\right)\right]\exp\left[\mathrm{i}\left(k_{z0}v_{z0}-\omega_0\right)t\right]\right\}$$
$$+\,\mathrm{c.c.}$$

$$v_y \cong \frac{\mathrm{i}q}{2m\gamma_0}\left\{\sum_{\substack{n=-\infty,\\ n\neq 0}}^{n=\infty}-\frac{\omega_{0n}'/u-v_{z0}k_{zn}'/u}{k_{xn}'\left(k_{zn}'v_{z0}-\omega_{0n}'\right)}d_{zn}g_n\left(v_{z0}t,t\right)\exp\left[\mathrm{i}\left(k_{xn}'x_r+\left\{k_{zn}'v_{z0}-\omega_{0n}'\right\}t\right)\right]\right.$$
$$+\frac{\left(v_{z0}/u\right)\tan\theta_{\mathrm{i}}-\sec\theta_{\mathrm{i}}}{k_{z0}v_{z0}-\omega_0}\left[d_{z01}g_{01}\left(v_{z0}t,t\right)\exp\left(\mathrm{i}k_{x0}x_r\right)\right.\quad\text{, (S1.16.2)}$$
$$\left.\left.-d_{z02}g_{02}\left(v_{z0}t,t\right)\exp\left(-\mathrm{i}k_{x0}x_r\right)\right]\exp\left[\mathrm{i}\left(k_{z0}v_{z0}-\omega_0\right)t\right]\right\}$$
$$+\,\mathrm{c.c.}$$

$$v_z-v_{z0} \cong -\frac{\mathrm{i}q}{2m{\gamma_0}^3}\left\{\sum_{\substack{n=-\infty,\\ n\neq 0}}^{n=\infty}\frac{1}{k_{zn}'v_{z0}-\omega_{0n}'}c_{zn}g_n\left(v_{z0}t,t\right)\exp\left[\mathrm{i}\left(k_{xn}'x_r+\left\{k_{zn}'v_{z0}-\omega_{0n}'\right\}t\right)\right]\right.$$
$$+\frac{1}{k_{z0}v_{z0}-\omega_0}\left[c_{z01}g_{01}\left(v_{z0}t,t\right)\exp\left(\mathrm{i}k_{x0}x_r\right)\right.\quad\text{, (S1.16.3)}$$
$$\left.\left.+c_{z02}g_{02}\left(v_{z0}t,t\right)\exp\left(-\mathrm{i}k_{x0}x_r\right)\right]\exp\left[\mathrm{i}\left(k_{z0}v_{z0}-\omega_0\right)t\right]\right\}$$
$$+\,\mathrm{c.c.}$$

$$x \cong \frac{q}{2m\gamma_0}\left\{\sum_{\substack{n=-\infty,\\ n\neq 0}}^{n=\infty}\frac{k_{zn}'-v_{z0}\omega_{0n}'/u^2}{k_{xn}'\left(k_{zn}'v_{z0}-\omega_{0n}'\right)^2}c_{zn}g_n\left(v_{z0}t,t\right)\exp\left[\mathrm{i}\left(k_{xn}'x_r+\left\{k_{zn}'v_{z0}-\omega_{0n}'\right\}t\right)\right]\right.$$
$$+\frac{\tan\theta_{\mathrm{i}}-\left(v_{z0}/u\right)\sec\theta_{\mathrm{i}}}{\left(k_{z0}v_{z0}-\omega_0\right)^2}\left[c_{z01}g_{01}\left(v_{z0}t,t\right)\exp\left(\mathrm{i}k_{x0}x_r\right)\right.\quad\text{, (S1.17.1)}$$
$$\left.\left.-c_{z02}g_{02}\left(v_{z0}t,t\right)\exp\left(-\mathrm{i}k_{x0}x_r\right)\right]\exp\left[\mathrm{i}\left(k_{z0}v_{z0}-\omega_0\right)t\right]\right\}$$
$$+\,\mathrm{c.c.}$$

$$y \cong \frac{q}{2m\gamma_0}\left\{\sum_{\substack{n=-\infty,\\ n\neq 0}}^{n=\infty} -\frac{\omega_{0n}'/u - v_{z0}k_{zn}'/u}{k_{xn}'\left(k_{zn}'v_{z0}-\omega_{0n}'\right)^2} d_{zn} g_n\left(v_{z0}t,t\right)\exp\left[\mathrm{i}\left(k_{xn}'x_r + \left\{k_{zn}'v_{z0}-\omega_{0n}'\right\}t\right)\right] \right.$$
$$+\frac{\left(v_{z0}/u\right)\tan\theta_\mathrm{i} - \sec\theta_\mathrm{i}}{\left(k_{z0}v_{z0}-\omega_0\right)^2}\left[d_{z01}g_{01}\left(v_{z0}t,t\right)\exp\left(\mathrm{i}k_{x0}x_r\right)\right. \quad , \qquad \text{(S1.17.2)}$$
$$\left.\left. - d_{z02}g_{02}\left(v_{z0}t,t\right)\exp\left(-\mathrm{i}k_{x0}x_r\right)\right]\exp\left[\mathrm{i}\left(k_{z0}v_{z0}-\omega_0\right)t\right]\right\}$$
$$+\,\mathrm{c.c.}$$

$$z - v_{z0}t \cong -\frac{q}{2m{\gamma_0}^3}\left\{\sum_{\substack{n=-\infty,\\ n\neq 0}}^{n=\infty} \frac{1}{\left(k_{zn}'v_{z0}-\omega_{0n}'\right)^2} c_{zn} g_n\left(v_{z0}t,t\right)\exp\left[\mathrm{i}\left(k_{xn}'x_r + \left\{k_{zn}'v_{z0}-\omega_{0n}'\right\}t\right)\right] \right.$$
$$+\frac{1}{\left(k_{z0}v_{z0}-\omega_0\right)^2}\left[c_{z01}g_{01}\left(v_{z0}t,t\right)\exp\left(\mathrm{i}k_{x0}x_r\right)\right. \quad , \qquad \text{(S1.17.3)}$$
$$\left.\left. + c_{z02}g_{02}\left(v_{z0}t,t\right)\exp\left(-\mathrm{i}k_{x0}x_r\right)\right]\exp\left[\mathrm{i}\left(k_{z0}v_{z0}-\omega_0\right)t\right]\right\}$$
$$+\,\mathrm{c.c.}$$

where the functions $g_\mathrm{n} = g_\mathrm{n}(x,z)$, $g_{01} = g_{01}(x,z)$, and $g_{02} = g_{02}(x,z)$ are as defined in Eq. (S3.6). “c.c.” refers to the complex conjugate of the expression it follows (recall that the coefficients e.g., c_zn, d_zn, are in general complex numbers, and that k_zn can be real or imaginary for different values of n). We have taken the liberty to ignore the exact value of the particle’s initial position in z, since it has negligible effect on the radiation spectrum, as long as the interaction spans more than a few metasurface periods.

Note that under the condition $\gamma \cong 1\big/\sqrt{1-\beta_z^2}$, which we have assumed, the momenta in the respective Cartesian directions may be approximated as $p_x = \gamma m v_x \cong \gamma_0 m v_x$, $p_y = \gamma m v_y \cong \gamma_0 m v_y$ and $p_z = \gamma m v_z \cong \gamma_0 m\left[v_z + \left(\gamma_0 v_{z0}/u\right)^2\left(v_z - v_{z0}\right)\right]$.

e Spectral intensity of emitted radiation

In this subsection, we solve for the radiation emission from of electrons interacting with the nanograting. We calculate the Liénard-Wiechert potentials, including the generalization to a medium where the speed of light is *u* instead of c, obtaining the energy distribution (specifically, energy radiated per unit solid angle per unit frequency) as

$$\frac{\mathrm{d}^2 I}{\mathrm{d}\omega \mathrm{d}\Omega} = \frac{q^2\omega^2}{16\pi^3}\sqrt{\frac{\mu}{\varepsilon}}\left|\mathbf{n}\times\mathbf{U}\right|^2 , \tag{S1.18}$$

where ε and μ are respectively the constant permittivity and permeability of the medium (so u = $(\varepsilon\mu)^{-1/2}$), $\mathbf{n} = (\sin\theta\cos\phi, \sin\theta\sin\phi, \cos\theta)$ is the unit vector in the direction of observation, as defined in Fig. 1, and the components of **U** are given by

$$U_i \equiv \int_{-\infty}^{\infty} \frac{v_i}{u} e^{\mathrm{i}\omega(t-\mathbf{n}\cdot\mathbf{r}/u)} , \tag{S1.19}$$

where ω is the frequency of observation. Carrying out the integral and ignoring higher-order terms, we obtain after some tedious algebra

$$\begin{aligned} U_x \cong \frac{\mathrm{i}q\sqrt{\pi/2}}{m\gamma_0 u\left(1-\sin\theta_\mathrm{i} v_{z0}/u\right)} \Bigg\{ & \sum_{\substack{n=-\infty,\\ n\neq 0}}^{n=\infty} \frac{k_{zn}' - v_{z0}\omega_{0n}'/u^2}{k_{xn}'\left(k_{zn}' v_{z0} - \omega_{0n}'\right)} c_{zn} \exp\left(\mathrm{i}k_{\mathrm{x}n}' x_r\right) G_n(\omega) \\ & + \frac{\tan\theta_\mathrm{i} - \left(v_{z0}/u\right)\sec\theta_\mathrm{i}}{k_{z0} v_{z0} - \omega_0}\left[c_{\mathrm{z}01}\exp\left(\mathrm{i}k_{\mathrm{x}0} x_r\right) G_+(\omega) - c_{\mathrm{z}02}\exp\left(-\mathrm{i}k_{\mathrm{x}0} x_r\right) G_-(\omega)\right] \Bigg\} \end{aligned} , \tag{S1.20.1}$$

$$\begin{aligned} U_y \cong \frac{\mathrm{i}q\sqrt{\pi/2}}{m\gamma_0 u\left(1-\sin\theta_\mathrm{i} v_{z0}/u\right)} \Bigg\{ & \sum_{\substack{n=-\infty,\\ n\neq 0}}^{n=\infty} -\frac{\omega_{0n}'/u - v_{z0} k_{zn}'/u}{k_{xn}'\left(k_{zn}' v_{z0} - \omega_{0n}'\right)} d_{zn} \exp\left(\mathrm{i}k_{\mathrm{x}n}' x_r\right) G_n(\omega) \\ & + \frac{\left(v_{z0}/u\right)\tan\theta_\mathrm{i} - \sec\theta_\mathrm{i}}{k_{z0} v_{z0} - \omega_0}\left[d_{\mathrm{z}01}\exp\left(\mathrm{i}k_{\mathrm{x}0} x_r\right) G_+(\omega) - d_{\mathrm{z}02}\exp\left(-\mathrm{i}k_{\mathrm{x}0} x_r\right) G_-(\omega)\right] \Bigg\} \end{aligned} , \tag{S1.20.2}$$

$$U_z \cong -\frac{\mathrm{i}q\sqrt{\pi/2}}{m\gamma_0 u\left(1-\sin\theta_\mathrm{i} v_{z0}/u\right)}$$

$$\cdot\left\{\sum_{\substack{n=-\infty,\\ n\neq 0}}^{n=\infty}\left[\frac{c_{zn}}{\gamma_0{}^2}+\frac{v_{z0}\left(\omega/u\right)}{k_{zn}'v_{z0}-\omega_{0n}'}\left(c_{zn}n_x\frac{k_{zn}'-v_{z0}\omega_{0n}'/u^2}{k_{xn}'}-d_{zn}n_y\frac{\omega_{0n}'/u-v_{z0}k_{zn}'/u}{k_{xn}'}-c_{zn}n_z\frac{1}{\gamma_0{}^2}\right)\right]\right.$$

$$\cdot\frac{1}{k_{zn}'v_{z0}-\omega_{0n}'}\exp\left(\mathrm{i}k_{xn}'x_r\right)G_n\left(\omega\right)$$

$$+\left[\left(\frac{c_{z01}}{\gamma_0{}^2}+\frac{v_{z0}\left(\omega/u\right)}{k_{z0}v_{z0}-\omega_0}\left\{c_{z01}n_x\left[\tan\theta_\mathrm{i}-\frac{v_{z0}}{u}\sec\theta_\mathrm{i}\right]+d_{z01}n_y\left[\frac{v_{z0}}{u}\tan\theta_\mathrm{i}-\sec\theta_\mathrm{i}\right]-c_{z01}n_z\frac{1}{\gamma_0{}^2}\right\}\right)\right.$$

$$\cdot\frac{1}{k_{z0}v_{z0}-\omega_0}\exp\left(\mathrm{i}k_{x0}x_r\right)G_+\left(\omega\right)$$

$$+\left(\frac{c_{z02}}{\gamma_0{}^2}-\frac{v_{z0}\left(\omega/u\right)}{k_{z0}v_{z0}-\omega_0}\left\{c_{z02}n_x\left[\tan\theta_\mathrm{i}-\frac{v_{z0}}{u}\sec\theta_\mathrm{i}\right]+d_{z02}n_y\left[\frac{v_{z0}}{u}\tan\theta_\mathrm{i}-\sec\theta_\mathrm{i}\right]+c_{z02}n_z\frac{1}{\gamma_0{}^2}\right\}\right)$$

$$\left.\left.\cdot\frac{1}{k_{z0}v_{z0}-\omega_0}\exp\left(\text{-}\mathrm{i}k_{x0}x_r\right)G_-\left(\omega\right)\right]\right\}$$

$$+\frac{v_{z0}T}{u}\operatorname{sinc}\left[\omega\left(1\text{-}\frac{v_{z0}}{u}n_z\right)\frac{T}{2}\right]$$

(S1.20.3)

The last term in (S1.20.3) corresponds to the Cherenkov radiation contribution of the charged particle moving through the medium. This term gives a negligible contribution in case the particle is slower than u (e.g., when in free space, u=c), as is expected from the Cherenkov effect. The functions $G_\pm\left(\omega\right), G_n\left(\omega\right)$ are defined as

$$G_n(\omega)=T_{0n}'\exp\left[-\left(\left\{k_{zn}'v_{z0}-\omega_{0n}'\right\}+\omega\left\{1-\frac{v_{z0}}{u}n_z\right\}\right)^2\frac{T_{0n}'^2}{2\left(1-\sin\theta_\mathrm{i}v_{z0}/u\right)^2}\right]$$

$$G_\pm(\omega)=T_0\exp\left[-\left(\left\{k_{z0}v_{z0}-\omega_0\right\}+\omega\left\{1-\frac{v_{z0}}{u}n_z\right\}\right)^2\frac{T_0{}^2}{2\left(1-\sin\theta_\mathrm{i}v_{z0}/u\right)^2}\right]\cdot \quad . \qquad \text{(S1.20.4)}$$

$$\exp\left[\mp\mathrm{i}\left(\left\{k_{z0}v_{z0}-\omega_0\right\}+\omega\left\{1-\frac{v_{z0}}{u}n_z\right\}\right)\frac{x_r\cos\theta}{u\left(1-\sin\theta_\mathrm{i}v_{z0}/u\right)}\right]$$

<u>Equations (S1.18) and (S1.20.1)-(S1.20.4) provide an analytical estimate</u> (semi-analytical if we obtain the c_z and d_z coefficients from the results of a numerical field solver) of the radiation intensity spectrum of a charged particle modulated by the fields of a plasmonic metasurface structure. We can see that the frequency peaks from the second-order scattering are located at

$$\omega_{\mathrm{pk},n} = \frac{\omega_{0n}' - k_{zn}' v_{z0}}{1 - \frac{v_{z0}}{u} n_z} . \tag{S1.21}$$

Note that depending on the value of n, the expression in Eq. (S1.21) can be positive or negative. Having a negative frequency is not a problem since the radiated field is real. The complex conjugate of each time harmonic exponential is also a constituent of the field, flipping the sign of the frequency. Its spectrum is thus conjugate symmetric and contains frequency peaks at *both* $\omega = \omega_{\mathrm{pk},n}$ and $\omega = -\omega_{\mathrm{pk},n}$, for each value of n. Nevertheless, negative frequencies still have an intriguing physical meaning. That the process can be understood as stimulated emission of the plasmon accompanying the (high frequency) photon emission. In contrast, the positive frequency means that the process can be understood as (stimulated) absorption of the plasmon accompanying the (high frequency) photon emission. This intriguing physics is analogous to the physics of the anomalous Doppler effect as explained by Ginzburg and Frank in [S1-S3].

In practice, for Eqs. (S1.20.1)-(S1.20.4), we only need to take into consideration one of each of these pairs of frequency peaks – the chosen one of each pair being given by Eq. (S1.21) – by definition of the single-sided intensity spectrum.

Equation (S1.21) also tells us that for nanostructures of relatively small period and electrons of relatively high speed such that $k_L v_{z0} >> \omega_0$, we should expect the single-sided spectrum to contain pairs of frequency peaks which are closely spaced in *absolute* frequency (separated by

$2\omega_0/(1 - v_{z0}n_z/u)$). Each of these pairs corresponds to peaks at n and at $-n$ (n being any integer except 0).

Our choice of a Gaussian profile for our input pulse in Eq. (S1.7) has led to a Gaussian shape for the spectral peaks in Eq. (S1.20.4). A different choice of input spectral profile only affects the shape of each radiative peak, without affecting key features like the peak frequency and spectral intensity of each peak.

f. The case of a continuous-wave laser

In the above, we have considered incident pulses that are finite in time, yet extended in space such that the interaction with the electron is always limited by the temporal extent of the pulses. In this subsection, we show that our analytical approach can also describe the opposite scenario. Here, the incident beams are finite in space, yet extended in time such that the interaction with the electron is always limited by the spatial extent of the beams. Comparing these two scenarios, we find that usually the only crucial parameter depicting the interaction result is the length (in time or space) of the interaction. Note that it can be substantially different in practice (e.g., the pulse case enables longer interactions for pulses moving close to the electron velocity, as we show in Figure 3 of the main text).

We consider the case of a (spatial) Gaussian beam exciting the metasurface. Therefore, the considered field is the Fourier decomposed E_x field at $x = x_r$ (Eq. S1.3) multiplied by a Gaussian envelope

$$E_{x0}(x,z,\omega_0)=\left\{\sum_{\substack{n=-\infty,\\ n\neq 0}}^{n=\infty} c_{xn}\exp[\mathrm{i}(k_{xn}x_r+nk_L z)]+c_{x01}\exp(\mathrm{i}k_{x0}x_r)+c_{x02}\exp(-\mathrm{i}k_{x0}x_r)\right\}\exp\left[-\frac{z^2}{2Z_0^2}\right], \tag{S1.22}$$

where Z_0 is related to the (intensity) full-width-at-half-maximum pulse duration Z_{FWHM} by the relation $Z_{\mathrm{FWHM}}=2\sqrt{\ln 2}T_0$. The other components rewrites similarly.

Operating again in the regime where $v_{x,y}/u$ << 1 and $\gamma\cong 1/\sqrt{1-\beta_z^2}\ \ \forall t$, metasurface we find that the velocity (through Newton-Lorentz equation of motion, see section *d*) are similar to equations (S1.16), replacing

$$g_n=g_{01}=g_{02}=\sqrt{2\pi}\,\frac{Z_0}{v_{z0}}\exp\left[-z^2/\left(2Z_0{}^2\right)\right], \tag{S1.23}$$

and $\delta\omega_n=0$, $\omega_{0n}=\omega_0$, $k_{xn}'=k_{xn}$ and $k_{zn}'=k_{zn}$.

Calculating the Liénard-Wiechert potential following the procedure in section *e*, we obtain the single-sided intensity spectrum as

$$\frac{\mathrm{d}^2 I}{\mathrm{d}\omega \mathrm{d}\Omega}=\frac{q^2\omega^2}{16\pi^3}\sqrt{\frac{\mu}{\varepsilon}}\left|\mathbf{n}\times\mathbf{U}\right|^2, \tag{S1.24}$$

where the components of **U** are given by

$$\begin{aligned}U_x\cong\frac{\mathrm{i}qZ_0\sqrt{\pi/2}}{m\gamma_0 u v_{z0}}&\left\{\sum_{\substack{n=-\infty,\\ n\neq 0}}^{n=\infty}\frac{k_{zn}-v_{z0}\omega_0/u^2}{k_{xn}\left(k_{zn}v_{z0}-\omega_{0n}\right)}c_{zn}\exp(\mathrm{i}k_{xn}x_r)G_n(\omega)\right.\\ &\left.+\frac{\tan\theta_{\mathrm{i}}-(v_{z0}/u)\sec\theta_{\mathrm{i}}}{k_{z0}v_{z0}-\omega_0}\left[c_{z01}\exp(\mathrm{i}k_{x0}x_r)-c_{z02}\exp(-\mathrm{i}k_{x0}x_r)\right]G_0(\omega)\right\}\end{aligned}, \tag{S1.25.1}$$

$$U_y \cong \frac{\mathrm{i}qZ_0\sqrt{\pi/2}}{m\gamma_0 u v_{z0}}\left\{\sum_{\substack{n=-\infty,\\ n\neq 0}}^{n=\infty} -\frac{\omega_0/u - v_{z0}k_{zn}/u}{k_{xn}'\left(k_{zn}'v_{z0}-\omega_{0n}'\right)} d_{zn}\exp\left(\mathrm{i}k_{\mathrm{x}n}x_r\right)G_n(\omega)\right.$$

$$\left. + \frac{\left(v_{z0}/u\right)\tan\theta_{\mathrm{i}} - \sec\theta_{\mathrm{i}}}{k_{z0}v_{z0}-\omega_0}\left[d_{\mathrm{z}01}\exp\left(\mathrm{i}k_{\mathrm{x}0}x_r\right) - d_{\mathrm{z}02}\exp\left(-\mathrm{i}k_{\mathrm{x}0}x_r\right)\right]G_0(\omega)\right\} \quad \text{, (S1.25.2)}$$

$$U_z \cong -\frac{\mathrm{i}qZ_0\sqrt{\pi/2}}{m\gamma_0 u v_{z0}}\exp\left[-Z_0{}^2 v_{z0}^2/2\right]$$

$$\cdot\left\{\sum_{\substack{n=-\infty,\\ n\neq 0}}^{n=\infty}\left[\frac{c_{zn}}{\gamma_0{}^2} + \frac{v_{z0}\left(\omega/u\right)}{k_{zn}'v_{z0}-\omega_{0n}'}\left(c_{zn}n_x\frac{k_{zn}'-v_{z0}\omega_{0n}'/u^2}{k_{xn}'} - d_{zn}n_y\frac{\omega_{0n}'/u - v_{z0}k_{zn}'/u}{k_{xn}'} - c_{zn}n_z\frac{1}{\gamma_0{}^2}\right)\right]\right.$$

$$\cdot\frac{1}{k_{zn}'v_{z0}-\omega_{0n}'}\exp\left(\mathrm{i}k_{\mathrm{x}n}'x_r\right)G_n(\omega)$$

$$+\left[\left(\frac{c_{z01}}{\gamma_0{}^2} + \frac{v_{z0}\left(\omega/u\right)}{k_{z0}v_{z0}-\omega_0}\left\{c_{z01}n_x\left[\tan\theta_{\mathrm{i}} - \frac{v_{z0}}{u}\sec\theta_{\mathrm{i}}\right] + d_{z01}n_y\left[\frac{v_{z0}}{u}\tan\theta_{\mathrm{i}} - \sec\theta_{\mathrm{i}}\right] - c_{z01}n_z\frac{1}{\gamma_0{}^2}\right\}\right)\right.$$

$$\cdot\frac{1}{k_{z0}v_{z0}-\omega_0}\exp\left(\mathrm{i}k_{\mathrm{x}0}x_r\right)$$

$$+\left(\frac{c_{z02}}{\gamma_0{}^2} - \frac{v_{z0}\left(\omega/u\right)}{k_{z0}v_{z0}-\omega_0}\left\{c_{z02}n_x\left[\tan\theta_{\mathrm{i}} - \frac{v_{z0}}{u}\sec\theta_{\mathrm{i}}\right] + d_{z02}n_y\left[\frac{v_{z0}}{u}\tan\theta_{\mathrm{i}} - \sec\theta_{\mathrm{i}}\right] + c_{z02}n_z\frac{1}{\gamma_0{}^2}\right\}\right)$$

$$\left.\left.\cdot\frac{1}{k_{z0}v_{z0}-\omega_0}\exp\left(-\mathrm{i}k_{\mathrm{x}0}x_r\right)\right]G_0(\omega)\right\}$$

$$+\frac{v_{z0}T}{u}\operatorname{sinc}\left[\omega\left(1-\frac{v_{z0}}{u}n_z\right)\frac{T}{2}\right]$$

(S1.25.3)

where

$$G_n(\omega) = \exp\left[-\left(\left\{k_{zn}v_{z0}-\omega_{0n}\right\} + \omega\left\{1-\frac{v_{z0}}{u}n_z\right\}\right)^2\frac{Z_0{}^2}{2v_{z0}{}^2}\right]. \quad \text{(S1.25.4)}$$

We can see that the frequency peaks from the second-order scattering are located at

$$\omega_{\mathrm{pk},n} = \frac{\omega_0 - k_{zn}v_{z0}}{1-\frac{v_{z0}}{u}n_z}. \quad \text{(S1.26)}$$

similar to Eq. (S1.21) for the pulsed version. The peak intensity (related to magnitude of the components **U**) is now scaled directly by the interaction length (controlled by Z_0) and no longer by matching the electron velocity with the pulse propagation embodied by the term $\left(1-\sin\theta_{\mathrm{i}} v_{z0}/u\right)$ (see discussion on Figure 3 in the main text).

S2. Comparison with *ab initio* calculations

This section presents comparisons between our analytical results (from Section S1) and our *ab initio* simulations. Altogether, we find a very good agreement.

Figure S2 shows that the analytical results agree well with the *ab initio* computation performed by the Classical ElectroDynamics and Radiation Integrated Computation (CEDRIC). The program solves the exact trajectories of the electrons with the Newon-Lorentz equation, based on the field computed by COMSOL at one frequency (we suppose first a dispersionless case to match the approximation in Eq. (S1.10)). The output spectrum radiated is computed by evaluating Eq. (S1.18). A slight discrepancy appears for high incident angle of the laser pulse ($\theta_i = 85°$ in Figure S2) because the higher order terms are neglected during the integration in Eq. (S1.19).

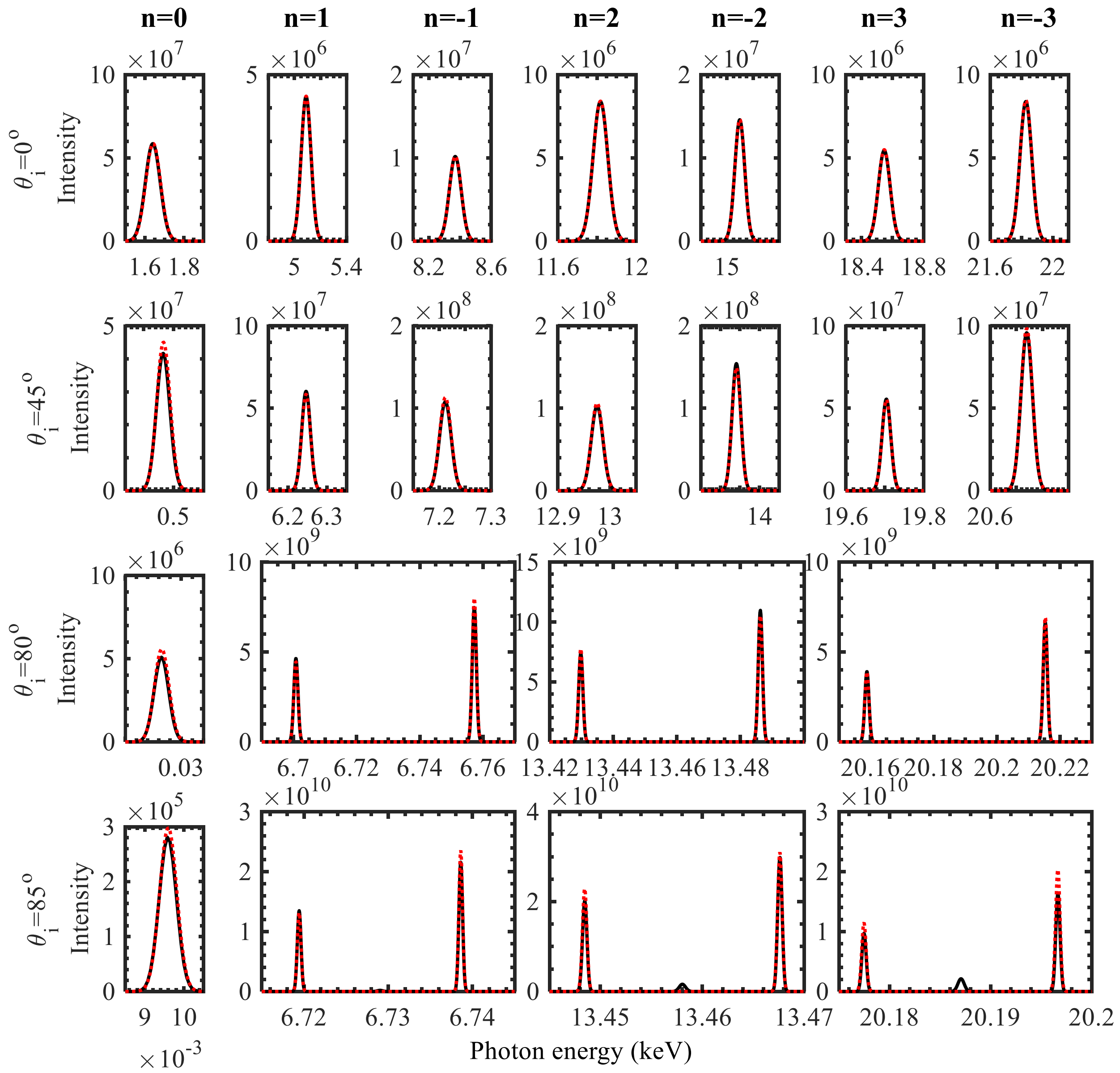

Figure S2: Comparison of the output spectrum between (dispersionless) *ab initio* calculations (solid black lines) and our analytical formulation (dashed red lines) for different incident angles of the laser pulse (θ_i). The laser wavelength is 370 nm with pulse duration of 10 fs and electron kinetic energy of 7.5 MeV. The metasurface is composed of three silver blocks with height of 40nm and widths 30, 7 and 25 nm at distances 10, 11 and 7 nm from each other, with a periodicity of 90 nm. The intensity is expressed in number of photons per second per steradian per 1% bandwidth.

In the main text, we consider a Gaussian pulse of T_{FWHM} = 10 fs (i.e. $T_0 = 6$ fs). Considering much shorter pulse durations will break the approximation in Eq. (S1.7) that is valid when $T_0 >> 2\pi / \omega_0 = 1\,\text{fs}$, and will then require a more complex analytical solution. Our numerical approach still work by solving for the metasurface frequency response to each frequency in the spectrum, then summing the different frequency components pondered with field amplitude $E_0 G(\omega)$ (Eq. (S1.9)) to generate the final output. When the structure reacts very differently to different frequencies (i.e., the structure's dispersion), we expect additional corrections. To quantify this correction, we compare in Figure S3 the dispersionless approximation (Eq. (S1.7)) with the *ab initio* computation considering the optical response of the silver metasurface at different frequencies. It shows that for the silver metasurface considered, the approximation holds with an error of maximum 1%. In fact, that approximation is legitimate when the dispersion of the material is relatively constant in the frequency region of interest. This is the case for silver around 370 nm, as it can be seen in Figure 4c in the main text. Note that for graphene nanoribbon array, the dispersion is extremely strong since the geometry alters the dispersion, creating narrow spectral features (due to the plasmonic nanoribbon mode). Considering a short pulse of a duration of 10 fs with the theory in this case would lead to an error of 2 orders of magnitude in the output emission spectrum, compared to the model including the dispersion (not shown here). This is why we consider a CW Gaussian beam on the graphene metasurface in Figures 1 and 4 in the main text.

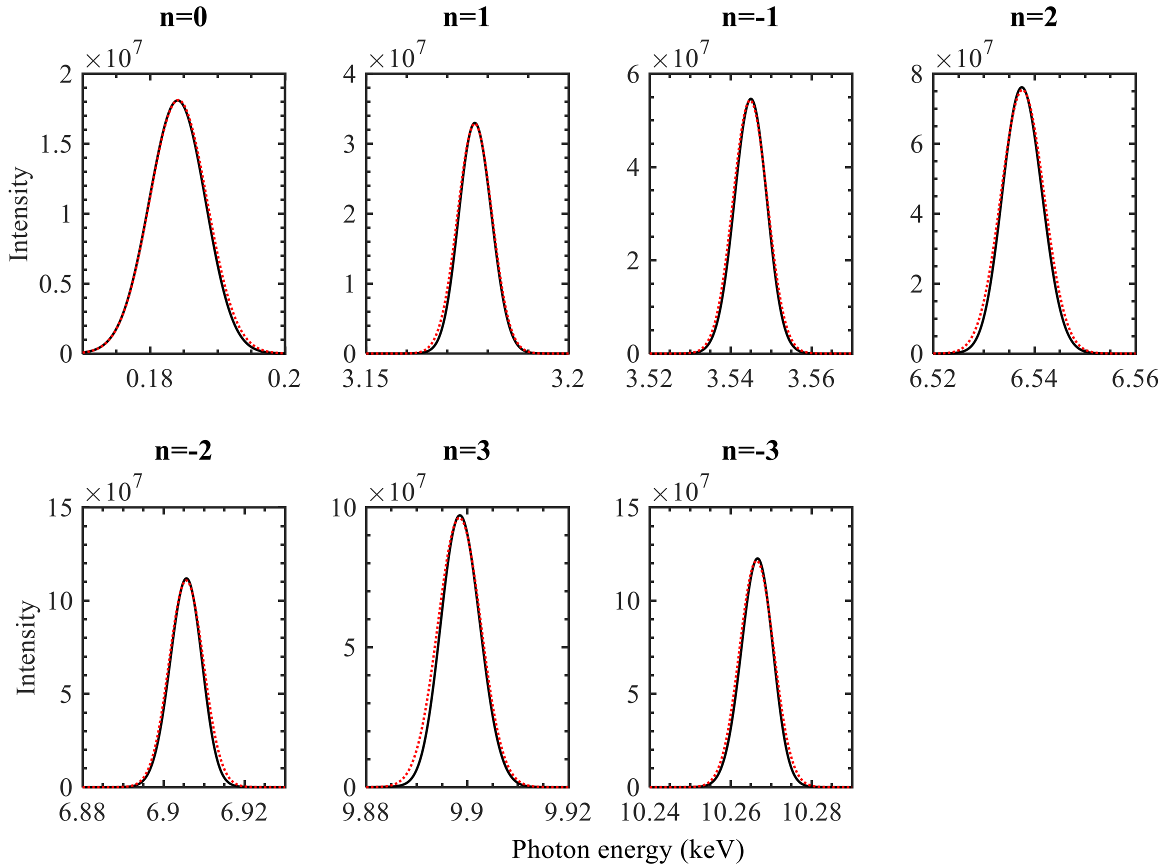

Figure S3: Comparison of the output spectrum between *ab initio* calculations (solid black lines) and our dispersionless analytical formulation (dotted red lines) for an incident angle of the laser pulse $\boldsymbol{\theta_i = 50°}$. The laser wavelength is 370 nm with pulse duration of 10 fs and electron kinetic energy of 5 MeV. The structure is the metasurface of Figure 1 in the main text.

S3. <u>Electron beam spatial extent</u>

In this section, we show that the solution based on the single electron can be used to predict the solution considering an electron beam of multiple electrons and a finite extent. We consider an elliptical beam of 100 µA current with focal plane widths of 3 nm in the x-direction and 3 µm in the y-direction. The beam is centered 1.5 nm away from the top of the metasurface, composed of silver blocks of 8.2 nm of 30 nm pitch, and illuminated with a 10 fs laser pulse at 60° incidence. In Figure S4, the radiation spectrum obtained with this setup is compared with the radiation emitted

from a single-electron shot 1 nm away from the same metasurface. In the two spectra, the peak frequencies and bandwidths are equal; the only discrepancy lies in the peak intensity, but this difference is well below an order of magnitude (maximum 13% in Figure S4). This difference is solely due to the range of electron distances from the grating (varying from 0 nm to 3 nm). Indeed, the space charge (inter-electron repulsion) is negligible for this range of parameters (being predicted to cause a beam divergence of $2.5 \cdot 10^{-12}$ rad after 1 mm of propagation according to [S4]). Interestingly, the error on the intensity is stronger for high orders (i.e. order 4 here). This behavior can be explained by the stronger field decay lengths of the high orders, as illustrated in Figure 5 of the main text.

We obtain a better match between the *ab initio* and the analytical calculations when considering 5-test-electron trajectories uniformly spaced apart. We suppose an elliptical beam of 100 µA current with focal plane widths of 5 nm in the x-direction and 3 µm in the y-direction. The beam is centered 3 nm away from the top of the metasurface (same as in Figure 6 in the main text), and illuminated by a 10 fs laser pulse at 50° incidence. The *ab initio* calculations are performed considering 200 electrons of 7.5 MeV energy and are represented in black solid lines in Figure S3. In order to obtain an analytical solution, we compute the spectrum generated by 5-test-electrons at various distances from the metasurface, ranging from 1 nm to 5 nm. The final spectrum is a weighted average of the obtained spectra, in order to take into account the elliptical shape of the beam in the y-direction. The solutions are plotted in red dashed lines in Figure S5, showing excellent agreement with the rigorous *ab initio* calculations. The parameters considered (for the metasurface and electron beam) are the same as Figure 5 in the main text.

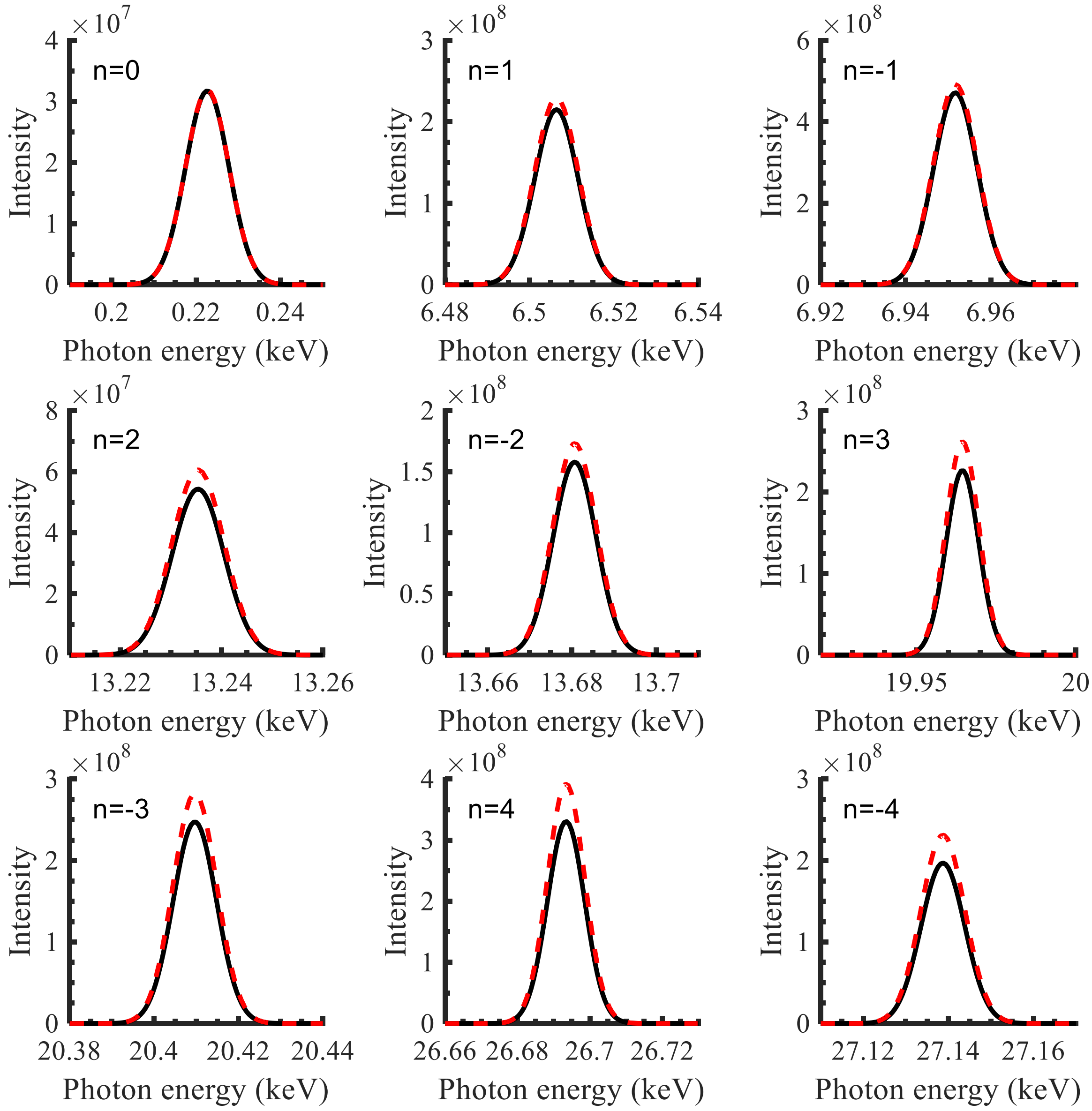

Figure S4: Electron beam (solid black) and single electron (red dashed) calculations lead to the same peak frequency and bandwidth, and a maximum difference of 10% in the predicted intensity, well within an order of magnitude. The elliptical electron beam has focal plane widths of 3 nm in the x-direction and 3 μm in the y-direction, is centered 1.5 nm from the metasurface (x-direction) and is composed of electrons of 5 MeV. The single electron at 7.5 MeV is shot 1 nm away from the metasurface. The metasurface is a periodic silver grating of 8.2 nm blocks arranged in a 30 nm period. The 10 fs optical pulse of wavelength 370 nm strikes the metasurface with an incidence angle of 60°. Intensity in units of photons/s/sr/1%BW.

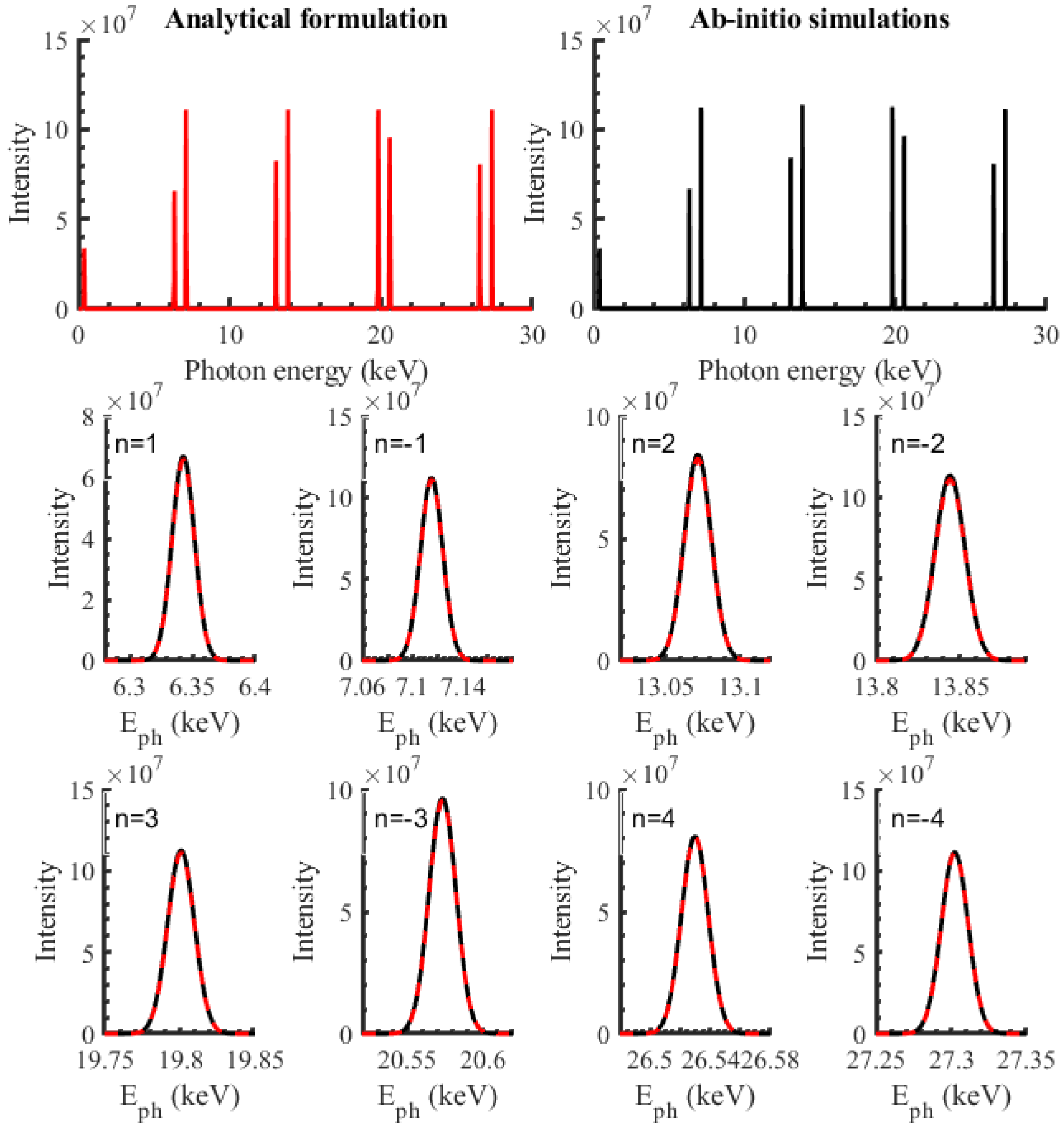

Figure S5: Excellent agreement between *ab initio* (black solid lines) and analytical predictions (red dashed lines). The elliptic electron beam has focal plane widths of 5 nm in the x-direction and 3 µm in the y-direction, is centered 3 nm from the metasurface (x-direction) and is composed of electrons of 7.5 MeV. The analytical formulation performs the weighted average of 5 spectra obtained considering independently 5-test-electrons at distance ranging from 1 nm to 5 nm from the metasurface.

S4. Electron beam angle dependence

In Figure 3 in the main text, we show the strong tunability of the peak brilliance and bandwidth with the angle of incidence of the photon beam. The key reason is that the electron-photon interaction is longer in time for a larger incidence angle. It is therefore also longer in space. From Eq. (S1.7), the spatial extent of the field on the metasurface is a Gaussian of spatial full-width-at-half-maximum (FWHM) (supposing $x_r = 0$ and $z = v_{z0}t$):

$$Z_{\mathrm{FWHM}} = \sqrt{2}\frac{v_{z0}T_{\mathrm{FWHM}}}{1-\beta\sin\theta_i} \quad \text{(S4.1)}$$

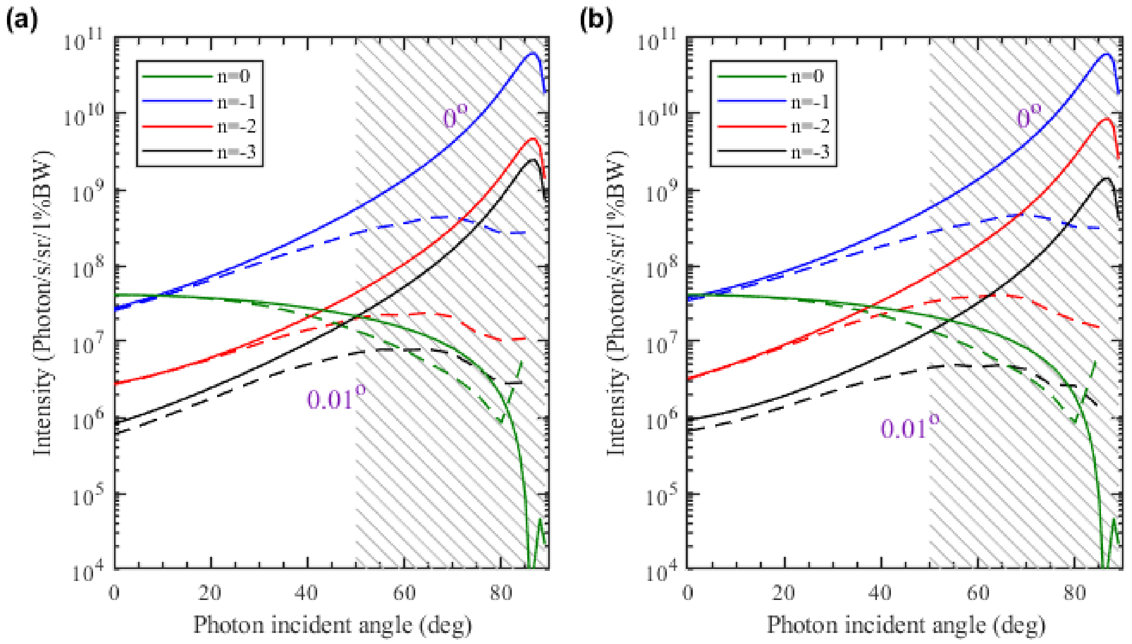

Figure S6: Effect of an electron traveling at an angle to the plane of the metasurface. The peak maximum brilliance for positive and negative orders are represented in (a) and (b), respectively, for an electron parallel to the metasurface (solid line) and with a deviation of 0.01° to the metasurface (dashed lines). The colors represent the orders: order 0 (green), order 1 (blue), order 2 (red) and order 3 (black). Electron energy is 7.5 MeV and the metasurface considered is the same as Figure 3 in the main text. The hatched area represents the region where the difference on the intensity resulting from the two different electron trajectories is more than 50%.

This dependence on the angle means that, for example with $\theta_i = 70°$, electrons of 7.5 MeV and T_{FWHM} = 10 fs the FWHM spatial extent of the Gaussian beam on the metasurface is $Z_{\mathrm{FWHM}} \approx 68$ µm. Therefore, if the electron is not correctly aligned, the electron-photon interaction will not be as efficient due to the exponential decay of the near field. For example, for a 0.01° deviation, after 68 µm in the z direction, it will present a deviation in the x direction of $\Delta x = 12$ nm, where the field intensity has decreased by a factor of 3. The impact of the deviation explains Figure S6, in which the maximum brilliance is reduced to $4.5 \cdot 10^8$ Photons/s/sr/1%BW (instead $6 \cdot 10^{10}$ Photons/s/sr/1%BW) and occurs at a smaller angle $\theta_i = 68°$ for the first order. In this case we synchronized the peak maximum of the pulse with the electron such that they meet 1 nm above the metasurface. Note that the simulations do not take into account the radiation generated by the metasurface due to the electron direct excitation (e.g., effects like Smith-Purcell radiation and bremsstrahlung).

S5. Laser fluence

This section studies the intensity limits on the incident laser due to the damage threshold of the structure. The brilliance of the source is a quadratic function of the amplitude of the field (proportional to $|c_{zn}|^2 |E_0|^2$, see Eqs. 3 and 4 in the main text). Therefore, in order to increase the intensity of the output radiation, one has to increase either the field enhancement (by engineering the metasurface), or the laser intensity (without damaging the metasurface). Here, we compute the fluence of the laser and we compare it to the fluence threshold that would damage the metasurface, based on developments in [30].

The fluence of our time-pulse Gaussian beam (Eq. S1.7) with field amplitude E_0 is

$$F_l = \int_{-\infty}^{\infty} \frac{c\varepsilon_0}{2} |E_0|^2 e^{-t^2/T_0^2} dt = \frac{c\varepsilon_0}{2} |E_0|^2 \sqrt{\pi} T_0 = \frac{1}{4}\sqrt{\frac{\pi\varepsilon_0}{\mu_0 \ln 2}} |E_0|^2 T_{\mathrm{FWHM}}, \qquad \text{(S5.1)}$$

In this article, we consider $T_{\mathrm{FWHM}} = 10$ fs and $E_0 = 1$ GV/m, leading to a fluence of $F_l = 1.4$ mJ cm^{-2}.

An estimation of the fluence threshold for structural damage is determined from the fluence required to reach the melting temperature of the silver metasurface, using [30]

$$F_{\mathrm{breakdown}} = mc_p \frac{T_{\mathrm{melt}} - T_0}{C_{\mathrm{abs}}}, \qquad \text{(S5.2)}$$

with m the mass of the silver metasurface, c_p the specific heat of the silver, C_{abs} the absorption cross-section, and T_{melt} and T_0 the melting temperature of the metal and the room temperature, respectively.

For example, for silver, $c_p = 0.233$ Jg^{-1}K^{-1}, $T_{\mathrm{melt}} = 1234$ K and density $\rho = 10.05$ g cm^{-3}. One period of the silver metasurface in Figure 2 has a cross-section surface of $2.6 \cdot 10^{-11}$ cm^{-2}. Supposing a transversal extent of 1 cm, the metal mass is $m = 2.73 \cdot 10^{-10}$ g. At normal incidence, the cross section is $C_{\mathrm{abs}} = 90 \cdot 10^{-7}$ cm^2. Therefore, at room temperature, the fluence damage threshold of the silver metasurface is $F_{\mathrm{breakdown}} = 6.6$ mJ cm^{-2}, which is above the laser fluence we considered.

S6. Asymmetry of the spectrum enabled by a blazed grating

Another way to design the output spectrum is the use of asymmetric metasurface building blocks. For example, in Figure S7, we show a simple periodic metasurface with trapezoidal elements. These structures will create an asymmetry between the negative and the positive orders of the near-field, resulting in an asymmetry in the pairs of peaks in the final spectrum. Note that $\theta_s = 0°$ at normal incidence gives a symmetry between negative and positive spatial orders, but there is still an asymmetry in the peak intensities, since the electron direction of motion breaks the symmetry.

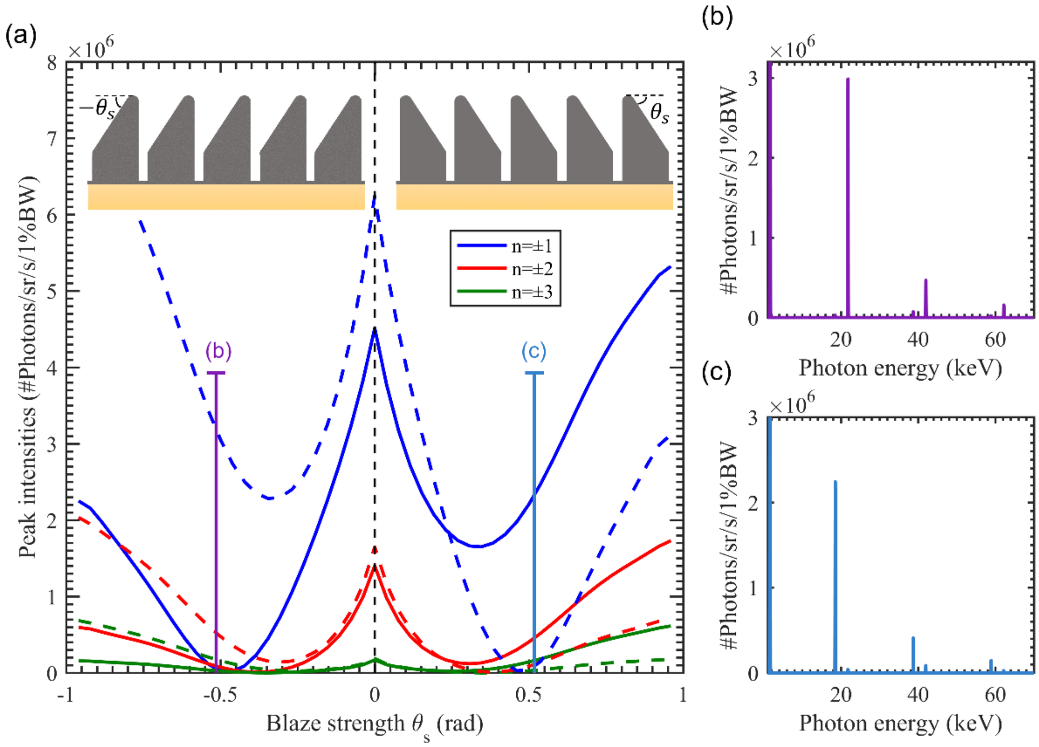

Figure S7: Tuning the relative intensity between positive (solid lines) and negative (dashed lines) orders. (a) The peak intensities for different slopes of the silver blocks ($\theta_s = 0$ rad is a rectangular block). The period is 30 nm, height is 40 nm, the pulse incidence angle is $\theta_i = 0°$ and the electron energy is 7.5 MeV. As examples, two complete spectra are represented for a blaze strength (b) $\theta_s = -0.6$ rad with a dominant negative order, and (c) $\theta_s = 0.6$ with a dominant positive order.

S7. DC fields

This section considers electron scattering off a DC metasurfaces, instead of the optical field considered above. Comparing the electron scattering by DC with the plasmonic near-fields in structures of a similar geometry shows both advantages and disadvantages. The advantage of biasing metallic structures, rather than employing an external laser excitation, is that it could reduce the fabrication complexity, especially for avoiding challenges of synchronizing and aligning a laser pulse with the trajectory and arrival times of electrons. However, the disadvantage is that DC fields will reduce the richness of the output spectrum, and limit the possibilities to tune the output radiation by altering the laser frequency.

Figure S8 shows a comparison between DC fields, with a voltage of 2.5 V (breakdown voltage of 20 nm graphene nanoribbons [S5]) and infrared fields of amplitude 0.1 GV m^{-1} (so the maximum field amplitude along graphene is 3 GV m^{-1}, below graphene breakdown threshold [31, 32]). Figure S9 shows the same comparison between the silver metasurface illuminated by an optical laser, and the same metasurface biased with the maximum breakdown voltage of 5 V, extrapolating the data in [S5] for a sub-10 nm gap in vacuum. In both Figures, the DC fields are computed with COMSOL, supposing a finite size of the metasurface (9.6 μm). This compares to the metasurface excited by a Gaussian beam forming a Gaussian spot with Z_{FWHM} = 9.6 μm on the metasurface and supposing an angle of incidence of 45°.

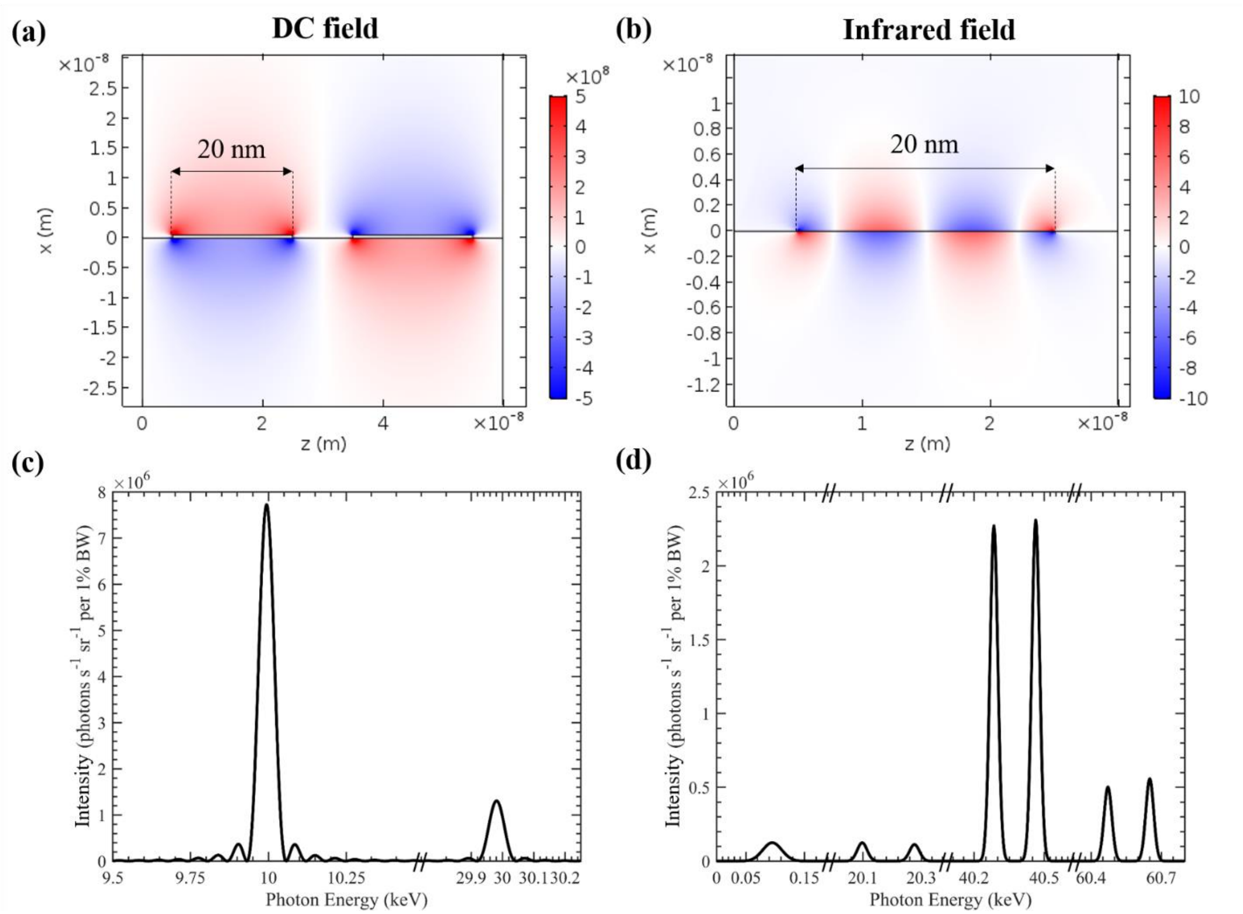

Figure S8: Comparison between a DC grating (left) and an infrared metasurface (right) realized using the same 20 nm wide (30 nm periodicity) graphene nanoribbon array. (a,b) Plotting the x-component of the electric field of the DC and infrared cases respectively, on a unit cell of (a) two and (b) one nanoribbon(s). (a) A positive voltage of 2.5 V (-2.5 V) is applied to the left (right) graphene nanoribbon in each unit cell, which creates an effective periodicity of 60 nm. (b) A 2 µm wavelength Gaussian beam with a field amplitude of 0.1 GV m^{-1} and a 45° angle of incidence excites primarily the second order mode of a graphene nanoribbons, which creates an effective periodicity of 15 nm (30 nm for the first order mode). (c,d) The corresponding X-ray spectra for the DC field from a metasurface of 9.6 µm (c) and for the infrared field from Gaussian spot of FWMH Z_{FWHM} = 9.6 µm on the metasurface (d). The energy of the electron is 7.5 MeV in both cases. In all cases, the the length of the graphene nanoribbon within each period is 20 nm.

A complete comparison of the DC and the optical Compton effects in metasurfaces. The resulting spectra (Figure S9) differ in the following ways:

- The 0th order does no longer appear with DC fields. The 0th order that corresponds to the incident plane wave does not contribute to the spectrum, since there is no external excitation by a plane wave.

- High orders do not appear in pairs anymore (± orders) in the DC field excitation. In contrast, the standing wave of the photonic fields can be seen as a sum of backward and forward propagating waves, giving rise to two scattering processes, and therefore two peaks. DC fields are static and therefore the two processes combine into a single peak.
- We find different peak profiles, caused by the different excitation methods. In the DC field excitation, the finite size of the metasurface generates sinc-like peaks. In the Gaussian laser pulse excitation, the spectrum has Gaussian peaks (see Section S1).
- The emission frequency is lower for DC fields, when supposing a mono-periodic structure (Figure S8). This arises from the negative and positive poles, doubling the effective period. Since the period is directly related to the emission frequency (see Eq. 1 in the main text), the output frequencies from DC mono-periodic metasurfaces are lower by a factor of two. Other metasurface designs, with a periodic base composed of more than one element, generally enable the poles to alternate faster, thus generating high harmonic emission in both the DC and optical excitation (with reduced brilliance of the high emission orders).

The main disadvantage of DC fields would be the diminished versatility of the design. Tuning the voltage remains the only dynamic parameter that can be controlled for a fixed metasurface in the DC case. Indeed, varying the voltage (as with the amplitude of the laser excitation) varies the brilliance of the source. In contrast, using an incident laser pulse allows one to directly control various aspects of the output radiation (such as brilliance, bandwidth and frequency) via the properties of the incident laser pulse such as its incidence angle (Figures 2 and 3 in the main text), the wavelength (Figure 4 in the main text) and the pulse duration. Another advantage of photonic structures over DC structures is the ability of the former to support higher order mode excitations, which can be engineered to further increase the X-ray

frequencies emitted without the need of smaller metasurface features. An example with second order plasmon mode in graphene nanoribbons is plotted in Figure S7b. In the graphene case, moreover, it can be shown that by changing either the wavelength of the laser source, or the doping, can decide whether the first (~ 20 keV) or second (~ 40 keV) order is dominant, further enhancing the versatility of the optically driven X-ray source.

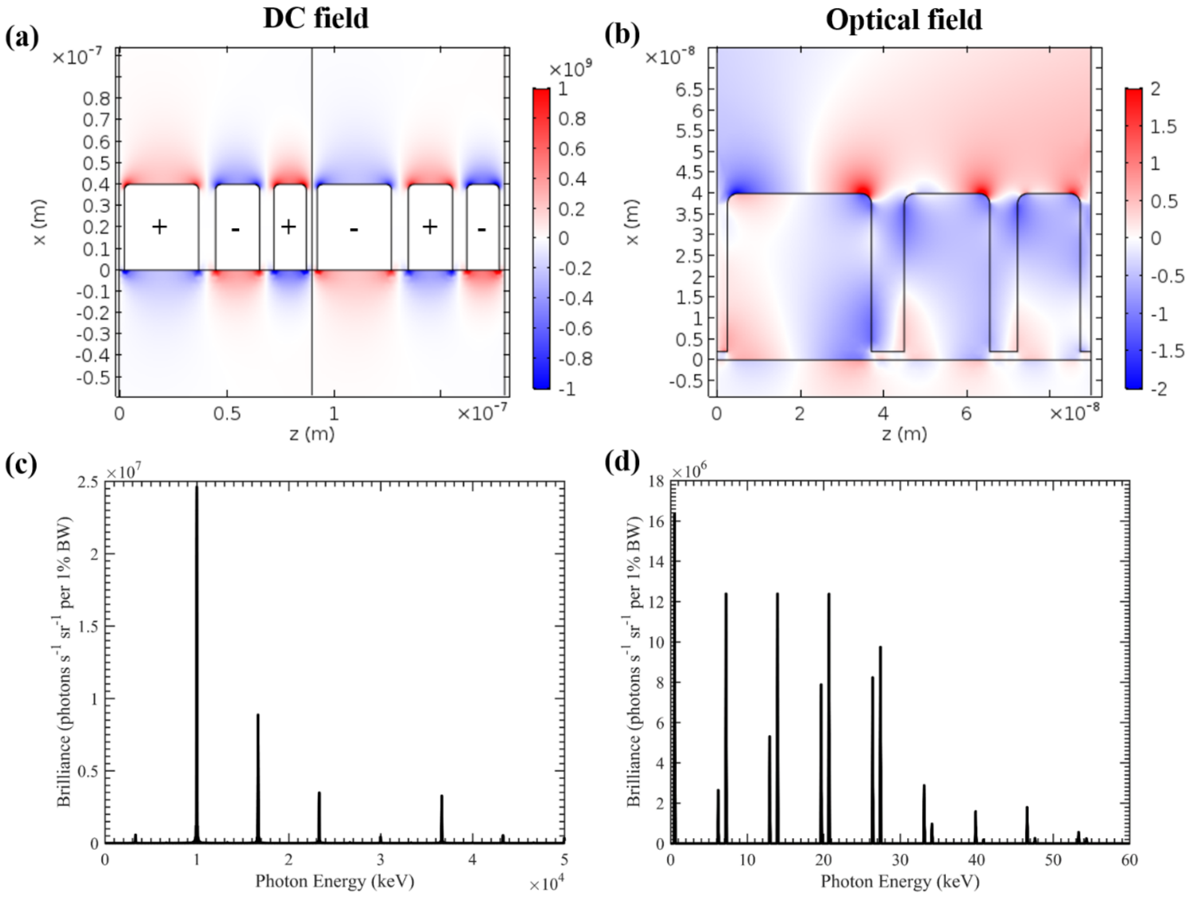

Figure S9: Comparison between DC grating (left) and optical metasurface (right) on a 40 nm thick (90 nm periodicity) silver metasurface. (a,b) Plotting the x-component of the electric field of the DC and optical cases respectively, on a unit cell of (a) two and (b) one period of the metasurface. An alternating positive and negative voltage of magnitude 5 V is applied as depicted by the signs, which creates and effective periodicity of 180 nm. (b) Plotting the x-component of the field enhancement when a 370 nm wavelength Gaussian beam with a field amplitude of 1 GV/m and a 45° angle of incidence excites the plasmons polaritons on top of the metasurface. (c-d) The corresponding X-ray spectra for the DC field from a metasurface of 9.6 µm (c) and for the optical field from Gaussian spot of Z_{FWHM} = 9.6 µm. The energy of the electron is 7.5 MeV in both cases.

References

[S1] I. M. Frank, "Optics of Light Sources Moving in Refractive Media," *Science,* vol. 131, pp. 702-712, 1960.

[S2] I. M. Frank and V. L. Ginzburg, *Dokl. Akad. Nauk SSSR*, vol. 56, no. 583, 1947.

[S3] V. L. Ginzburg, "Reviews of Topical Problems: Certain Theoretical Aspects of Radiation due to Superluminal Motion in a Medium," *Soviet Physics Uspekhi,* vol. 2, pp. 874-893, 1960.

[S4] L. J. Wong, I. Kaminer, O. Ilic, J. Joannopoulos and M. Soljacic, "Towards graphene plasmon-based free-electron infrared to X-ray sources," *Nat Photon,* vol. 10, pp. 46--52, 2015.

[S5] R. M. Meindl, Y. Yang, K. Brenner, T. Beck and J. D., "Breakdown current density of graphene nanoribbons," *Appl. Phys.,* vol. 94, p. 243114, 2009.

[S6] G. Meng, Y. Cheng, K. Wu and L. Chen, "Electrical Characteristics of Nanometer Gaps in Vacuum under Direct Voltage," *IEEE Transactions on Dielectrics and Electrical Insulation,* vol. 21, p. 4, 2014.
